# Construction and Diversification of Natural Product Biosynthetic Gene Clusters at High Efficiency and Accuracy

**DOI:** 10.1021/acssynbio.5c00601

**Published:** 2025-10-10

**Authors:** Chaoxian Bai, Lina M. Bayona, Gilles P. van Wezel

**Affiliations:** Institute of Biology, 4496 Leiden University, Sylviusweg 72, 2333 BE Leiden, Netherlands

**Keywords:** biosynthetic gene cluster, Golden Gate Assembly, natural product biosynthesis, actinorhodin, *Streptomyces*, synthetic biology

## Abstract

Biosynthetic gene clusters (BGCs) encode the biosynthesis of natural products, which serve as the foundation for therapeutics such as antibiotics, anticancer agents, antifungals, and immunosuppressants. The vast majority of the BGCs remain uncharacterized due to lack of expression or inability to cultivate the native host, making refactoring and expression of BGCs in optimized hosts a prerequisite for genome-based drug discovery. Transformation-associated recombination (TAR) cloning and Gibson assembly are error prone due to the use of homologous recombination. Here, we present a BGC cloning and refactoring strategy based on a hierarchical Golden Gate Assembly (GGA), which enables systematic pathway engineering and mutagenesis with unprecedented accuracy and efficiency. We constructed the 23 kb actinorhodin (ACT) BGC and 23 mutant derivatives with either one of the *act* genes inactivated, within the same experiment and with 100% efficiency. Introduction of the BGCs in the ACT-nonproducer *Streptomyces coelicolor* M1152 revealed that nine genes are essential for ACT production, while inactivation of others led to significant rewiring of the biosynthetic pathway. Global Natural Products Social (GNPS) molecular networking thereby revealed a surprisingly large number of unidentified molecules, significantly expanding the chemical space associated with ACT biosynthesis. Additionally, we refactored the *act* cluster through promoter engineering and evaluated expression outcomes across multiple *Streptomyces* strains. Together, our work establishes a GGA-based platform for BGC construction, refactoring, and functional dissection, accelerating synthetic-biology-driven natural product discovery.

## Graphical abstract

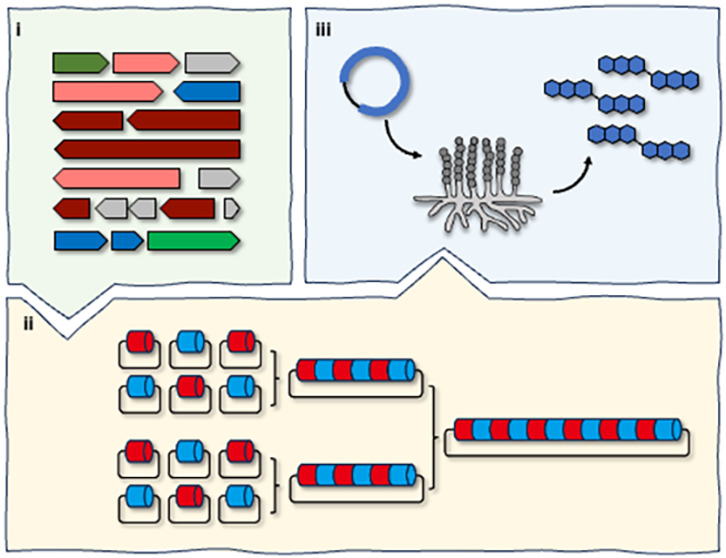

## Introduction

Natural products derived from diverse sources—such as plants and microorganisms—have long been indispensable in antibiotic discovery.^1^ Traditionally, high-throughput screening of extensive strain and compound libraries has been central to identifying bioactive molecules.^2,3^ However, this approach often leads to the rediscovery of known compounds and overlooks many biosynthetic gene clusters (BGCs) that remain transcriptionally silent under standard laboratory conditions.^4^ Additionally, a significant portion of microbial biodiversity remains inaccessible due to the inability to culture certain strains or the lack of genetic tools to manipulate them.^5,6^

To overcome these limitations, the field has increasingly shifted toward genome-guided discovery, enabled by advances in DNA sequencing technologies since the early 2000s. These developments have illuminated the vast biosynthetic potential encoded within microbial genomes, facilitating the identification of BGCs and their associated metabolic products. Yet despite progress in genome mining tools capable of predicting natural product biosynthetic pathways,^7,8^ many BGCs remain functionally uncharacterized due to poor expression or inaccessibility in their native hosts.^9,10^ To fully unlock this hidden metabolic potential, there is a growing need for high-throughput and error-free BGC cloning and expression platforms. Such technologies would allow systematic, scalable reconstruction of biosynthetic pathways in tractable heterologous hosts, offering a powerful route to access novel natural products and expand nature’s chemical repertoire for therapeutic and industrial applications.

A major technical hurdle in this process is the cloning of BGCs, which are often large (over 10 kb), GC-rich, and composed of multiple genes that must be accurately captured and maintained within a suitable vector. Methods such as the RecET system^11^ in *Escherichia coli* and transformation-associated recombination (TAR)^12^ cloning in yeast have been effectively used to clone BGCs by leveraging endogenous recombination systems. Additionally, CRISPR-based technologies have been developed to enable precise excision and assembly of target BGCs into destination vectors.^13–15^ Once the BGCs are successfully cloned, the next critical step involves modifying the BGCs through techniques such as gene deletion, insertion, or pathway refactoring. These modifications aim to enhance the production of the desired metabolites or enable the synthesis of novel compounds. Techniques like CRISPR/Cas9 and TAR-mediated recombination are widely employed to specifically modify BGCs *in vitro*, including promoter engineering to activate silent BGCs transcriptionally.^16–20^ The development of CRISPR tools further enables precise *in situ* BGC editing, allowing for single nucleotide substitutions^21^ and the exchange of subdomains within complex megasynthase assembly lines.^22^ Despite their success, these methods are often time-consuming and labor-intensive, limiting their practicality for large-scale parallel engineering. An important drawback is that the high sequence similarity within many BGCs, especially those encoding polyketide synthases (PKS) and nonribosomal peptide synthetases (NRPS), poses challenges for homologous recombination-based techniques, increasing the risk of off-target effects and unintended recombination events.

Golden Gate Assembly (GGA) offers a powerful alternative for modular and high-fidelity DNA assembly. Owing to its efficiency, scalability, and ability to assemble multiple DNA fragments in a predefined order within a single reaction, GGA has become a routine and indispensable tool in synthetic biology. It has been widely used in the construction of genetic circuits, where precise arrangement of promoters, coding sequences, and terminators is essential.^23^ Recently, GGA has been successfully applied to reconstruct larger gene clusters, including polyketide synthase (PKS)^24^ and nonribosomal peptide synthetase (NRPS)^25^ biosynthetic gene clusters, nitrogen fixation gene clusters,^26^ and even a complete phage genome.^27^ These advances highlight the versatility of GGA as a universal platform for the rapid and reliable construction of large DNA assemblies. Nevertheless, compared to direct cloning strategies such as CATCH^13^ and TAR cloning,^12^ applications of GGA for the assembly and refactoring of entire BGCs remain relatively limited. Its efficiency and robustness in handling large and complex BGCs have not yet been fully demonstrated, which may have led to an underestimation of its potential. With further optimization, GGA could become a transformative strategy to meet the increasing demand for natural product discovery and pathway engineering, enabling the systematic exploration of cryptic BGCs and the expansion of chemical diversity.

In this study, we harnessed GGA for the assembly and refactoring of BGCs, capitalizing on its scarless assembly, automation compatibility, and avoidance of homologous recombination—features that make it well-suited for large-scale BGC engineering. Our strategy enables hierarchical *de novo* assembly of BGCs from manageable short fragments, substantially increasing the flexibility and efficiency of BGC manipulation, as exemplified by single-nucleotide resolution editing and promoter engineering (Scheme 1). To demonstrate its effectiveness, we assembled, mutagenized, and refactored the 23 kb actinorhodin (ACT) BGC from *Streptomyces coelicolor*, showcasing the potential of this approach for the precise and scalable construction of complex BGCs in microbial natural product discovery and development.

**Scheme 1:**
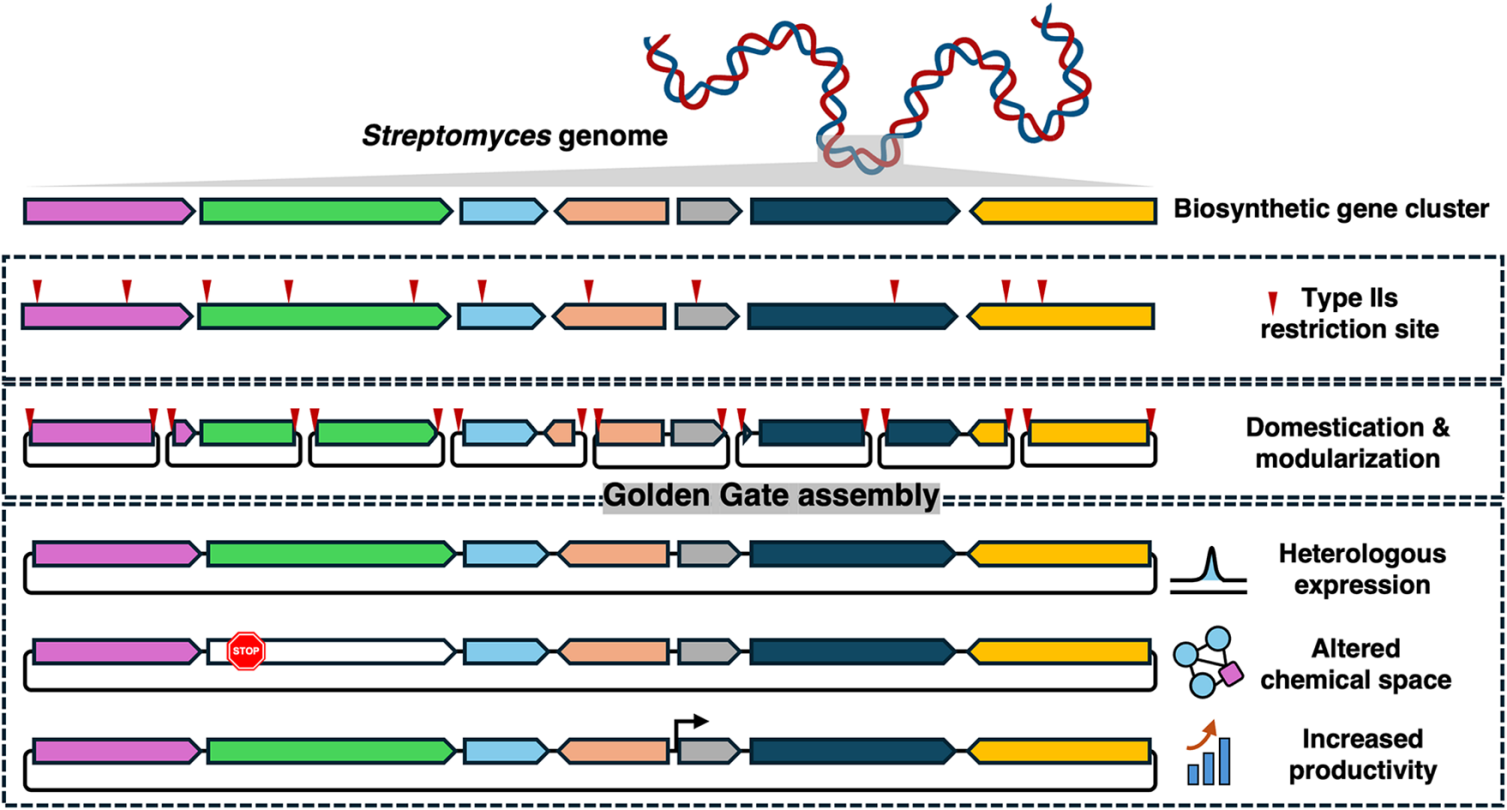
Golden Gate Assembly Strategy for *De Novo* Construction and Diversification of Natural Product Biosynthetic Gene Clusters (BGCs)[Fn s1fn1]

a
*Streptomyces* genomes often contain dozens of BGCs. To enable efficient assembly and systematic refactoring, type IIs restriction sites within a target BGC are first removed, and the cluster is fragmented for subcloning into entry vectors. These modularized BGC fragments provide a versatile platform for *de novo* reassembly of the entire cluster. The fragmented architecture also facilitates targeted modifications, such as the introduction of premature stop codons or replacement with synthetic promoters. This *de novo* assembly strategy supports heterologous expression, expands access to alternative chemical space, and enhances production of desired natural products.

## Results

### *De Novo* Strategy for Biosynthetic Gene Cluster Assembly

In microbial genomes, BGCs typically consist of several to dozens of genes, covering up to 100 kb or more of DNA. Due to the considerable size and intricate organization of BGCs, we employed a strategic bottom-up methodology that systematically reconstructs modular segments, functionally analyzes key genes and biosynthetic steps, and enables efficient heterologous expression in optimized microbial hosts.

As the basis for the technology, we built further on the well-established method of Golden Gate assembly (GGA).^28^ As one of the most widely used DNA assembly techniques, GGA facilitates simultaneous assembly of multiple DNA fragments in a predetermined order *in vitro*. Leveraging this approach, our goal is to establish a streamlined pipeline for BGC engineering—from cloning through to refactoring. By minimizing the need for iterative trial-and-error, our approach enhances the reproducibility and efficiency in the construction of complex genetic architectures. This platform lays the groundwork for the accelerated discovery and optimization of natural products through programmable pathway design.

We chose the actinorhodin (ACT) BGC from *S. coelicolor* as the test system to develop the methodology, as it is a well-known model for polyketide biosynthesis. In addition, the blue-pigmented ACT can be readily detected, thus providing a convenient visual readout. The *act* cluster comprises 23 genes organized in six operons and is contained within a 23 kb DNA segment.^29,30^ To enable GGA, the recognition sites for the two type IIS restriction enzymes employed (BsaI and PaqCI) were eliminated in a process known as domestication, which involves the removal of internal restriction sites (Figure 1A). Out of the 25 BsaI and 3 PaqCI restriction sites, 26 reside within the coding sequence, which can be removed through silent mutations. The remaining two BsaI sites in noncoding regions were altered by converting G to C or C to G, respectively (Figure S1 and Table S5). Assembly junction sequences were selected based on the predicted sets of high-fidelity four-base overhangs.^31^ Additionally, to ensure long-term storage and facilitate future experiments, the short DNA fragments were subcloned into the entry vector pKan^32^ to enhance their stability and accessibility. Instead of segmenting the BGC based on gene content, we subcloned individual DNA fragments into approximately 2 kb segments, streamlining the cloning process and simplifying confirmation through Sanger sequencing.

**Figure 1:**
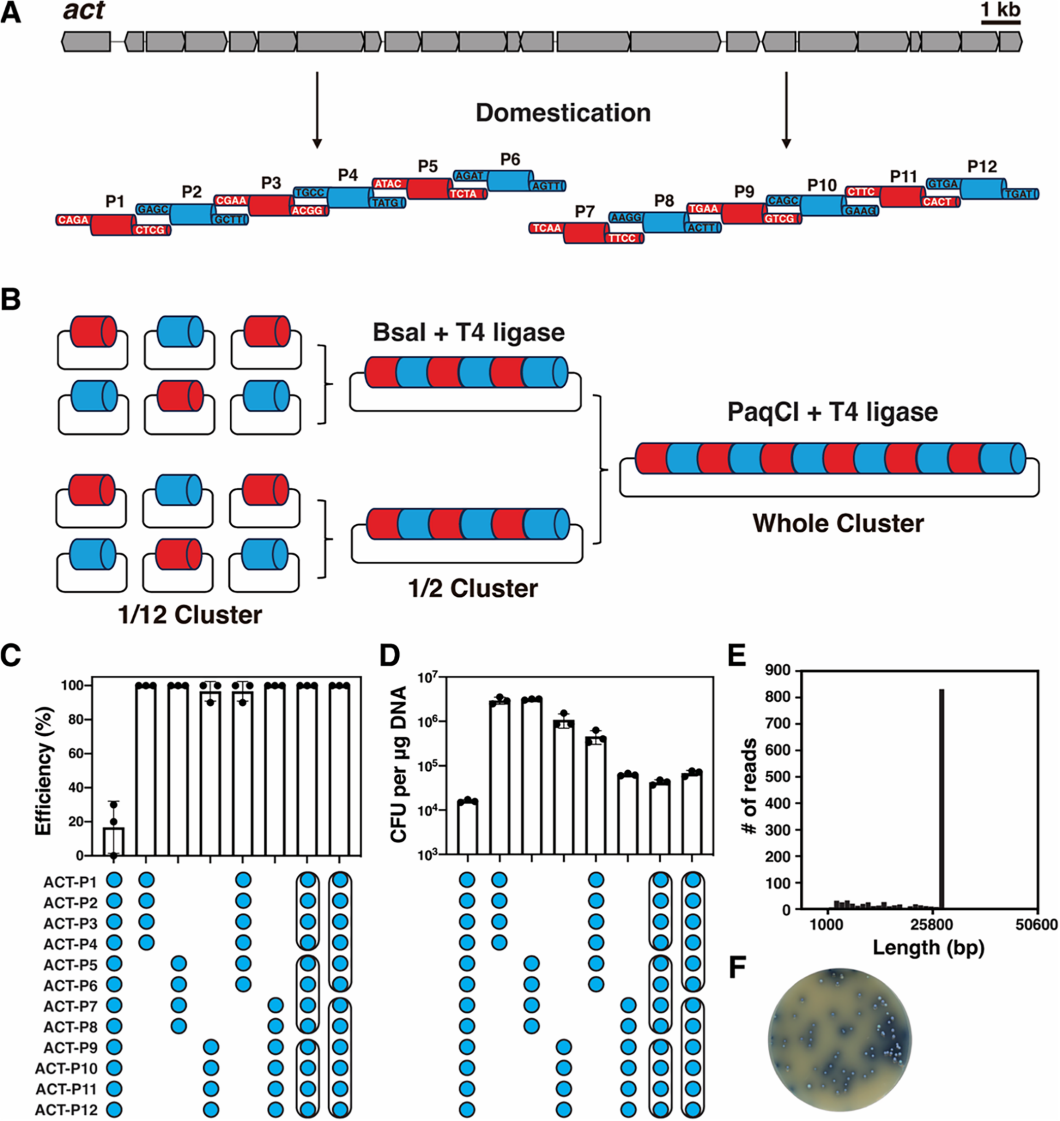
Efficient and precise *de novo* assembly of the actinorhodin biosynthetic gene cluster. (A) Illustration depicting the fragmentation and domestication of a 23 kb *act* gene cluster into 12 segments, each with a unique 4-bp overhang. (B) Utilization of a two-step hierarchical GGA approach to assemble the complete BGC from individual fragments subcloned in entry vectors. (C) bar chart representing the efficiency of GGA of 12 *act* parts, either through one-pot assembly or a two-step hierarchical approach. Black dots indicate the results obtained from ten randomly selected colonies, which were further validated through *Bam*HI digestion or colony PCR. (D) High efficiency electroporation-mediated transformation of *E. coli* with GGA ligation mixture. Error bars, ±1 SD (E) Verification of assembled plasmid containing the intact *act* gene cluster using Oxford Nanopore Technologies (ONT). The dominant peak in the histogram signifies the presence of a plasmid containing the *act* gene cluster, measuring 28,167 bp in length. (F) Following conjugation into the *act*-deficient heterologous host *S. coelicolor* M1152, the reassembled *act* cluster successfully restored blue pigmentation on soy flour mannitol (SFM) plates.

### Highly Efficient Assembly of the *act* Cluster

In order to facilitate the rapid and efficient assembly of the complete 23 kb *act* cluster in one go, we first combined all 12 subcloned fragments with the destination vector pPAP-RFP-BsaI in a single GGA reaction. Successful assembly of the *act* cluster was verified by a *Bam*HI restriction analysis. However, this one-pot reaction resulted in less than 20% of the transformants harboring the entire *act* cluster (Figure 1C). For the *lac* operon cassette, a higher efficiency was reported,^33^ but this cassette is much smaller (4.9 kb). Clearly, the probability of mismatched base pairs increases with size due to the addition of more assembly junction sequences, resulting in truncated assemblies that may outcompete the desired construct during transformation.

To further improve the GGA efficiency, a multilevel hierarchical approach was implemented, this time using a two-step process (Figure 1B). First, less than 10 entry plasmids harboring *act* cluster fragments were assembled into the intermediate vector pAmp-RFP-BsaI in a reaction mixture containing BsaI-HFv2 and T4 ligase. Subsequently, two or three intermediate plasmids underwent a secondary GGA into the destination vector pPAP-RFP-PaqCI in a reaction containing PaqCI and T4 ligase (Figure 1B). This multilevel hierarchical approach achieved a nearly 100% assembly efficiency for up to six fragments (Figure 1C). Additionally, the multilevel approach achieved significantly higher transformation efficiency, exceeding that of the one-pot assembly method by at least 10-fold (Figure 1D). The assembled *act* cluster was further subjected to nanopore sequencing, demonstrating the reliability of the GGA approach (Figure 1E). Given that substantial effort is required to verify the correct assembly, particularly when dealing with multiple BGCs simultaneously, the hierarchical GGA methodology guarantees a much higher success rate, offering great potential for streamlining the manipulation of BGCs in a high-throughput manner. The assembled *act* cluster was conjugated into the heterologous expression host *S. coelicolor* M1152, which lacks four endogenous BGCs including *act*.^34^ Indeed, *S. coelicolor* M1152 harboring the reassembled *act* cluster restored ACT production, as evidenced by the blue pigment secreted into the SFM agar plate (Figure 1F). This indicates that subtle modifications to remove type IIS restriction sites within the *act* cluster are well-tolerated.

### Exploring the Roles of Biosynthetic Genes in the *act* Cluster via Stop Codon Mutagenesis

To further assess the effectiveness and advantages of our *de novo* strategy for BGC assembly, the biosynthetic pathway of ACT was investigated further via targeted modification. Research into ACT biosynthesis began over four decades ago, leading to the identification of the full set of genetic information necessary for its production.^29^ Subsequent studies focused on individual genes within the BGC, often employing traditional methods such as gene deletion to elucidate their functions in the biosynthetic pathway. However, these deletions can cause polar effects on adjacent gene expression, complicating result interpretation and potentially disrupting the overall gene context. By leveraging smaller subcloned BGC fragments, which are more amenable to precise manipulation, specific sections of the cluster or even individual nucleotides can be targeted with greater accuracy and efficiency.

To get full insights into the impact of individual *act* gene mutations on the ACT-related chemical space, we applied the technology for site-directed mutagenesis, introducing a stop codon into each gene within the *act* cluster (Figure 2A). This resulted in 23 mutant *act* clusters, each with a single gene producing a prematurely terminated protein, thereby losing its function (Figure 2B). The wild-type cluster and its 23 mutant derivatives were transformed into *S. coelicolor* M1152. Visual assessment revealed that of the all 23 transformants expressing one of the mutant BGCs, 12 were still blue pigmented. This indicates that 12 out of 23 *act* genes are not required *per se* for ACT production. Among the remaining 11 genes, six showed altered pigmentation, indicating modified biosynthesis pathways; transformants carrying BGCs with mutations in *act*VI-ORF1 or ORF2 produced pink pigments, those with BGCs mutated in *act*VI-ORF3 were purple, those with mutations in *act*VA-ORF4 or ORF5 were brown-pigmented, and an *act*III mutant BGC led to production of a light orange pigment. The other five clones, mutant for *act*II-ORF4, *act*I-ORF1-ORF3, or *act*VII, did not produce any detectable pigments on R5 or SFM agar plates (Figures 2C and S4). It is noteworthy that our initial attempt to mutate the *act*I-ORF1 gene (encoding KSα that is required for ACT biosynthesis) surprisingly did not affect ACT production. On closer inspection, we discovered a downstream TTG codon, which we propose is the true translational start codon, thereby resulting in a protein that is 43 amino acids shorter. Supporting this hypothesis, AlphaFold structural prediction of the annotated gene reveals a low-confidence region within the N-terminal extension before the TTG codon. In addition, transcription start site (TSS) mapping indicates that the TSS lies downstream of the previously annotated start codon,^35^ further supporting that translation is initiated from the downstream TTG codon (Figure S5). Notably, while ATG is the most commonly preferred start codon, alternative codons such as GTG and TTG are also utilized for translation initiation, complicating accurate gene annotation in *Streptomyces*.^35,36^

**Figure 2:**
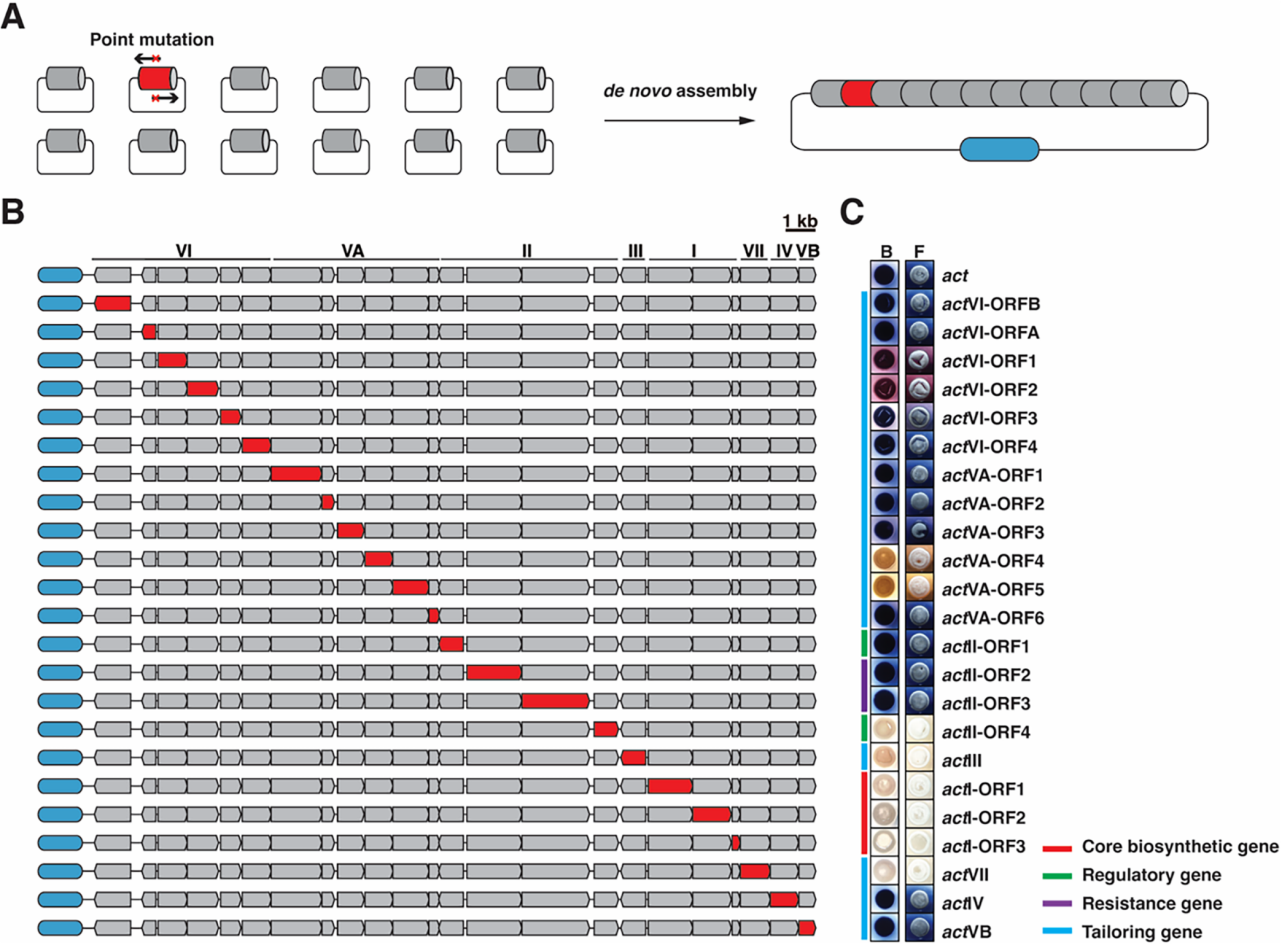
Stop codon scanning mutagenesis of 23 biosynthetic genes (SCO5070–5092) in the *act* gene cluster. (A) Point mutations were carried out via site-directed mutagenesis on target fragments in the *act* gene cluster subcloned in the entry plasmid. The blue rounded rectangle represents the destination vector pPAP. (B) Architecture of the *act* gene cluster and its 23 single-gene mutant derivatives, each carrying a premature stop codon inserted after the start codon in the gene highlighted in red. The genes shown in gray remain unchanged, matching the wild-type *act* cluster. (C) Phenotypes of *S. coelicolor* M1152 harboring the *act* gene cluster or one of its 23 single-gene mutant derivatives grown on R5 agar. First row, M1152 harboring the wild-type *act* cluster; rows 2–24, M1152 harboring either of the 23 derivative *act* clusters. Colonies were grown for 4 days on R5 agar. F, front of colony; B, back of colony.

### Metabolomics Analysis of the *act* Mutants and Novel Insights into the Biosynthetic Pathway

The ACT biosynthetic pathway involves key steps, such as the assembly of the core carbon skeleton by the type II minimal PKS, followed by sequential modifications including ketoreduction, oxidation, and dimerization. Figure 3A illustrates the currently proposed biosynthetic pathway of actinorhodin, adapted from previous studies.^30,37,38^ To gain a comprehensive understanding of the ACT biosynthetic pathway, metabolomics was performed. For this, *S. coelicolor* M1152 transformants harboring either the wild-type BGC or one of the mutant derivatives were grown on R5 agar plates for 4 days, after which the biomass and the agar were extracted using ethyl acetate. The extracts were analyzed using liquid chromatography combined with mass spectrometry (LC-MS). The resulting data were further processed with MZmine 3 and MetaboAnalyst 6.0. Principal component analysis (PCA) was performed to assess the similarity of metabolomic profiles among the *S. coelicolor* M1152 strains harboring *act* clusters (Figure S6). Expectedly, the PCA plot revealed that mutants producing the blue pigment clustered closely with the wild-type *act* cluster, while the four colorless nonproducers (BGC mutants *act*I-ORF1–3 and *act*II*-*ORF4) separated along the PC1. Additionally, mutants associated with tailoring genes involved in later biosynthetic stages as well as those encoding ketoreductase (*act*III) and aromatase (*act*VII), also formed a separate, more dispersed cluster.

**Figure 3:**
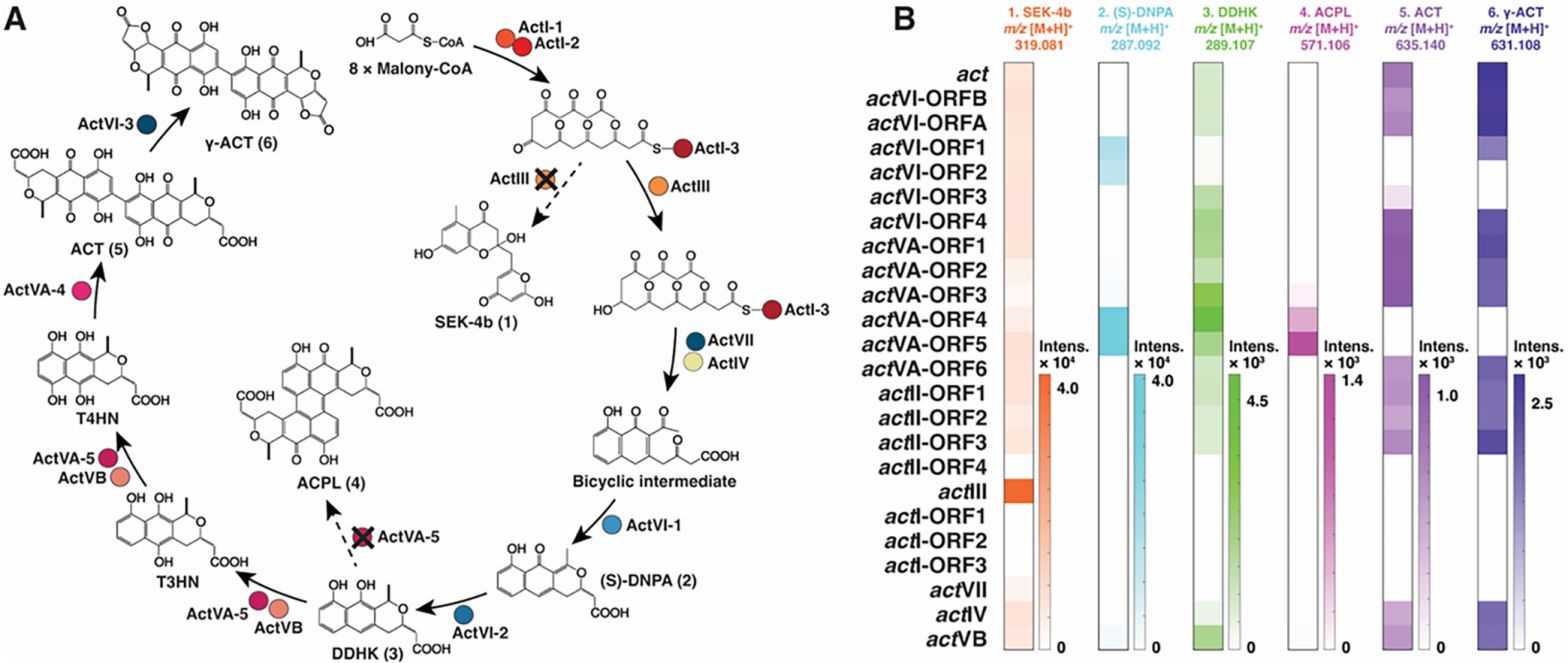
Metabolomic analysis of *S. coelicolor* M1152 harboring either the wild-type *act* cluster or one of its mutant derivatives. (A) Currently proposed biosynthetic pathway for ACT. Dashed arrows indicate the shunt pathway. (B) Heatmap displaying the relative abundance of ACT **5** and γ-ACT **6**, along with its two intermediates ((S)-DNPA, **2** and DDHK, **3**) and two shunt products (SEK-4b, **1**, and ACPL, **4**) within the pathway. Values shown are averages of three biological replicates.

To visualize the metabolomic signatures for each BGC derivative, features corresponding to known metabolites in the ACT biosynthetic pathway were further investigated in detail to better understand these shifts (Figures 3B and S7). The shunt product SEK-4b **1** was detected in M1152 harboring wild-type or any of the 19 *act* mutant clusters that showed pigmentation but was absent in the four colorless nonproducers *act*I-ORF1–3 and *act*II-ORF4. **1** accumulated especially highly in M1152 with the *act*III mutant derivative, consistent with ActIII as the ketoreductase responsible for reducing the carbonyl group at C-9.^39^ The intermediates (S)-DNPA **2** and DDHK **3** accumulated most prominently in *S. coelicolor* M1152 with the *act*VA-ORF4 mutant cluster. In contrast, the brown shunt product ACPL **4** showed the highest accumulation in M1152 with *act*VA-ORF5 mutant BGC and was slightly enriched in strains with *act*VA-ORF3,4 and *act*VB mutant clusters. This conforms well to studies on the functional roles of essential enzymes (*act*VA-ORF3–5 and *act*VB) involved in the later stages of ACT biosynthesis.^30,37^ The final products, ACT **5** and its lactonized derivative γ-ACT **6**, were detected in extracts from transformants expressing the wild-type cluster and 14 out of 23 mutant clusters. Notably, although strains harboring the *act*VI-ORF1 and *act*VI-ORF3 mutant clusters exhibited altered pigmentation on R5 agar plates, their extracts showed the presence of actinorhodins at reduced levels.

To get a more detailed overview of the new chemical space that may be produced by the different recombinants, we utilized Global Natural Products Social (GNPS) molecular networking.^40^ To focus specifically on ACT-related compounds, metabolites originating from the chassis strain *S. coelicolor* M1152—lacking the *act* cluster—were filtered out. Structurally related metabolites were then grouped into molecular families, enabling a deeper investigation into the diversity of derivatives arising from ACT biosynthetic pathways. In addition to previously characterized products, molecular networking revealed a complex and diverse chemical landscape associated with these pathways (Figure S8). Notably, many detected metabolites could not be matched to known compounds, indicating the presence of previously uncharacterized chemical entities. Surprisingly, more than half of these metabolites were absent in the wild-type ACT producer, suggesting that they are not synthesized by the native *act* cluster. While the major molecular families could be associated with ACT-related compounds, including intermediates and shunt products, the network also revealed several smaller clusters and singletons of uncertain origin. It is unclear whether these features arise from unrelated biosynthetic pathways, spontaneous chemical modifications, or cryptic metabolic activities. Notably, GNPS-based annotation identified two non-ACT metabolites, Desferrioxamine E and futalosine, which were highly enriched in nonproducer strains carrying inactive *act* clusters, specifically in mutants disrupted at *act*I-ORF1–3 and *act*II-ORF4 (Figure S8).

Importantly, our analysis also redefines the role of *act*IV. While *act*IV has long been considered the second-ring cyclase in the pathway, our data show that it is not strictly indispensable. In the absence of ActIV, the pathway can still proceed; however, instead of committing to the bicyclic intermediate, the system yields 3-hydroxy-6-(2-methyl-4-oxochromen-5-yl)-5-oxohexanoic acid, a nascent aromatic polyketide scaffold (Figure S8). This assignment was supported by MS/MS fragmentation patterns consistent with those reported in the GNPS database (Figure S9). This discovery suggests that ActIV plays a crucial role in guiding the correct cyclization pattern. Nonetheless, the presence of these unknown analogs significantly expands the chemical space associated with the ACT pathway, revealing a far greater metabolite diversity than previously recognized.

### Refactoring and Heterologous Expression of the *act* Cluster in Diverse *Streptomyces* Strains

BGC refactoring, such as promoter engineering, is a powerful synthetic biology tool for activating, fine-tuning, and optimizing the production of natural products. We here employed our new BGC cloning strategy to refactor the *act* cluster, by including different synthetic promoters with increasing strength—SP2, SP5, SP10, SP15, SP20, and SP26—to modulate constitutive ACT production by activating *act*II-ORF4 (Figure 4A).^41^ For this, six synthetic promoter-RBS combinations were inserted upstream of *act*II-ORF4 in entry plasmid pKan-*act*12.8 and subsequently assembled into a refactored *act* cluster. The six refactored *act* clusters along with the wild-type cluster were transformed into *S. coelicolor* M1152 and three other heterologous hosts, namely a derivative of *Streptomyces lividans* 1326 that lacks its native *act* cluster, *Streptomyces venezuelae* ATCC15439, and *Streptomyces roseosporus* ATCC31568. After cultivation in R5 liquid medium for 4 days, total ACT (actinorhodin and γ-actinorhodin) production was quantified by measuring the OD_640_ of the cultures after KOH treatment (Figure 4B). In *S. coelicolor*, the different synthetic promoters resulted in varying levels of ACT production depending on their strength, with only SP5 surpassing the production levels of the wild-type promoter. Although blue pigmentation was observed on SFM agar plates after introduction of the refactored *act* cluster into *S. lividans* and *S. venezuelae*, ACT production remained negligible in liquid-grown R5 cultures (Figures 4B and S10). Interestingly, the presence of *act* clusters with *act*II-ORF4 under the control of promoters SP10, SP15, SP25, or SP26 inhibited aerial hyphae formation in *S. roseosporus* transformants, likely as a trade-off for increased ACT production (Figure S10). The host-dependent variability in ACT production underscores the critical roles of both the promoter strength driving the SAPR family regulator ActII-ORF4 and the host genetic background in the success of BGC refactoring strategies.

**Figure 4:**
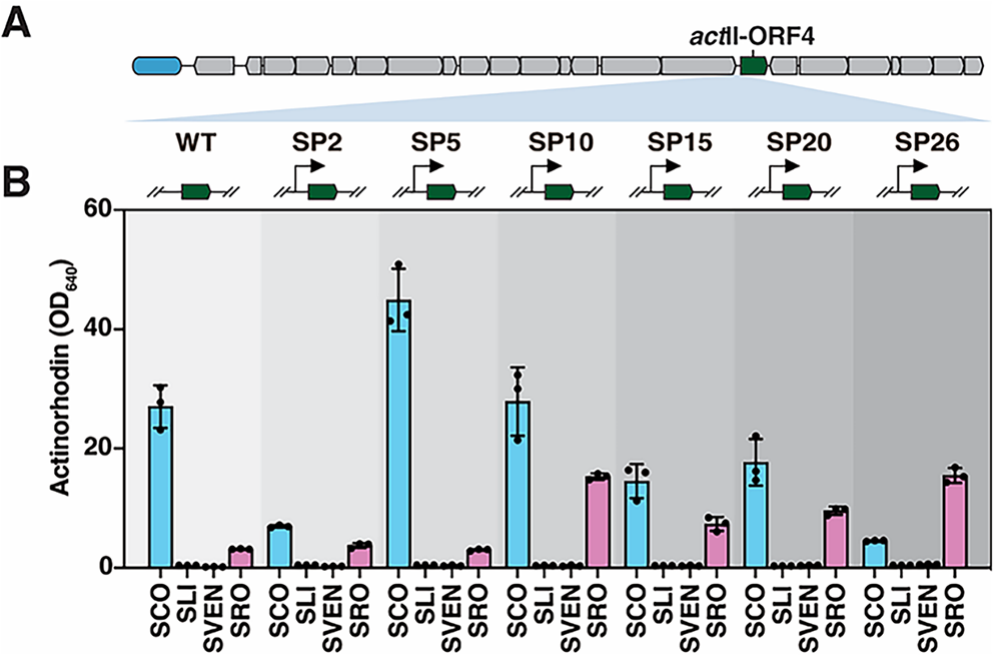
Refactoring of the *act* gene cluster using synthetic promoters and heterologous expression in various *Streptomyces* hosts. (A) Six synthetic promoters (SP2, SP5, SP10, SP15, SP20, SP26) with different strengths were inserted upstream of the SARP famliy regulatory gene *act*II-ORF4. (B) Wild-type and six refactored *act* gene clusters were introduced into four *Streptomyces* species, and ACT production quantified by measuring the optical density at 640 nm. SCO, *S. coelicolor* M1152; SLI, *S. lividans* 1326Δ*act*_*SL*_; SVEN, *S. venezuelae* ATCC15439, SRO, *S. roseosporus* ATCC31568. Error bars, ±1 SD.

## Discussion

In this study, we present a robust technology platform for BGC cloning and engineering that offers superior advantages over traditional homologous recombination-based cloning strategies (Table S7). While recombination methods rely on sequence homology, they are frequently hindered by similar or repetitive regions within large BGCs—particularly in modular PKS/NRPS that harbor extensive repeats—thereby necessitating *in-silico* redesign and *de novo* gene synthesis to eliminate direct repeats.^20^ In contrast, our platform utilizes Type IIS restriction enzymes to carry out high-fidelity digest-and-ligate reactions, generating unique, user-defined overhangs that ensure precise fragment orientation—features strongly supported by previous studies.^27,31,33^ By using high-fidelity four-base overhang sets, correct Watson–Crick pairings are formed in the assembly, which assures the efficiency and accuracy of the GGA-based approach.^31^ Importantly, because fragment joining is guided solely by these overhangs, the GGA approach is unaffected by internal repeat sequences within the BGCs.

This modular and hierarchical system drastically reduces errors and assembly failures, maximizing the success rate of each build. Once individual building blocks are domesticated and stored in entry vectors, downstream assemblies can proceed without further PCR amplification, minimizing the risk of introducing mutations and streamlining quality control. The error-proof workflow significantly simplifies the construction and modification of BGCs, regardless of their size or genetic complexity. Its scalability and compatibility with iterative design-build-test cycles make it especially advantageous for applications in synthetic biology and metabolic engineering. Furthermore, the use of prevalidated modules eliminates the need to resequence entire constructs, accelerating the overall engineering workflow and making it highly compatible with automation and high-throughput platforms. In this work, we focused on the *act* cluster as a model system. While relatively modest in size, the hierarchical design of the GGA platform enables iterative rounds of assembly, allowing seamless expansion to much larger constructs. With this strategy, final assemblies can readily reach 100 kb or more,^42^ demonstrating the scalability of the approach for handling even the largest and most complex BGCs.

A bottleneck of using GGA for BGC engineering lies in the need to eliminate internal Type IIS restriction sites—a process referred to as domestication. Given the size of BGCs, dozens of these sites are often present and must be removed before assembly. This step therefore represents a challenge when applying GGA to native gene clusters, especially at high throughput. Potential solutions include identifying novel Type IIS enzymes that recognize rarer sequences or leveraging synthetic gene services to redesign and synthesize DNA fragments free of restriction sites. As DNA synthesis becomes increasingly cost-effective,^43^ these barriers are expected to diminish, further solidifying GGA as a powerful platform for BGC refactoring and natural product discovery.

Our hierarchical assembly strategy was employed successfully to systematically refactor the entire 23-gene *act* cluster from *S. coelicolor*. By engineering targeted gene disruptions across individual biosynthetic and tailoring genes, a comprehensive library of derivative BGCs was obtained, each carrying a single gene inactivated through the introduction of a premature stop codon. This enabled the interrogation of gene essentiality and function within the pathway, generating a detailed functional map of the *act* cluster. The metabolomics analysis provided new insights into the ACT biosynthetic pathway. Despite decades of study, new pieces of the biosynthetic jigsaw are still being revealed.^30,44,45^ As anticipated, derivatives lacking either the core minimal PKS gene (*act*I-ORF1–3) or the pathway-specific SARP regulator (*act*II-ORF4) failed to produce ACT or any related metabolites. Beyond these essential components, several tailoring enzymes also proved indispensable, namely the ketoreductase ActIII, the cyclase ActVII, enzymes mediating redox modifications (ActVI-ORF2 and ActVA-ORF5), and the dimerization enzyme ActVA-ORF4. A recent study demonstrated that ActVI-ORF3 plays a critical role in lactonization of ACT to γ-ACT.^46^ Our findings are consistent with this, as only ACT but not γ-ACT was detected in the ActVI-ORF3 mutant derivative. The role of ActVB in ACT biosynthesis has been subject to debate; while some studies reported it as essential,^21,47^ others showed that ActVB mutants still produce ACT.^38^ This discrepancy can be explained by the activity of ActVA-ORF6, which is capable of catalyzing the same reaction as that of the ActVA-ORF5/ActVB system. Our findings support the latter view, highlighting the presence of functional redundancy in the pathway.

Systematic perturbations of the pathway uncovered unexpected functional redundancies and bottlenecks while simultaneously generating BGC variants that accumulated novel biosynthetic intermediates and structural analogs absent in the wild-type strain. A striking example was observed in the *act*IV mutant; while its gene product was previously defined as the second-ring cyclase, our MS/MS analysis of the mutant’s extracts identified the accumulation of the shunt product 3-hydroxy-6-(2-methyl-4-oxochromen-5-yl)-5-oxohexanoic acid, a nascent aromatic polyketide scaffold. The emergence of such metabolites substantially expands the chemical space associated with ACT biosynthesis and demonstrates how BGC refactoring can expose cryptic or alternative metabolic outputs. Collectively, these findings showcase the utility of GGA for reconstructing and refactoring complex BGCs, providing a robust platform to dissect biosynthetic logic and unlock chemical diversity. We note, however, that MS/MS-based molecular networking does not afford full structural elucidation, and the exact structures of the many newly detected metabolites need to be resolved using, among others, NMR.

Refactoring the *act* cluster through promoter engineering further demonstrated the capacity of the platform to modulate the pathway activity with precision. By introducing synthetic promoters of varying strengths upstream of *act*II-ORF4, encoding the pathway-specific activator, we established a tunable system for ACT production. In *S. coelicolor* M1152, ACT output varied with promoter strength, with SP5 yielding the highest levels, surpassing the wild-type promoter. These results emphasize the importance of balanced activation—demonstrating that moderate expression can outperform both weak and overly strong promoter-driven systems and underlining the necessity of fine-tuning regulatory inputs even within familiar hosts to achieve optimal metabolic output. In heterologous hosts, ACT production was highly context dependent. While blue pigmentation on solid media confirmed pathway activation in *S. lividans* and *S. venezuelae*, ACT production in liquid cultures remained minimal. These results point to the influence of media conditions and host-specific factors on metabolite biosynthesis and underscore the need for tailored optimization strategies in heterologous expression systems. *S. roseosporus*, in contrast, exhibited a complex but overall a more favorable response. Although strong promoter constructs inhibited aerial hyphae formation, likely due to metabolic burden or regulatory interference, ACT production in liquid cultures was more robust. This favorable performance may reflect the evolutionary proximity of *S. roseosporus* to *S. coelicolor*—close enough to ensure functional compatibility of the refactored BGC, yet genetically distinct enough to avoid repressive interactions with native regulatory systems. Similar trends have been observed using chassis-independent genome engineering strategies such as CRAGE, where intermediate phylogenetic distance from the native host promoted enhanced metabolite diversity and yield by alleviating regulatory constraints.^48^

The synthetic promoters used in this work have been benchmarked previously using reporter genes such as sfGFP, with relative strengths showing strong cross-species correlation^41^ and additional validation in cell-free systems.^49^ However, ACT represents a more complex case: beyond functioning as an antibiotic, it is also a redox-active small molecule capable of influencing cellular physiology and fitness in ways unrelated to its antimicrobial function.^50^ As a result, robust promoter sets that perform predictably with reporters do not necessarily yield reliable outputs in the context of secondary metabolite biosynthesis. This discrepancy reflects the fact that reporter assays capture transcriptional activity in isolation, whereas metabolite production is influenced by a broader network of regulatory, metabolic, and physiological factors. Taken together, these findings emphasize that host selection, promoter strength, and genetic background act in concert to determine the final metabolite output.

In summary, this work establishes a versatile and modular framework for BGC engineering, providing a rapid, precise, and scalable solution for reconstructing and functionally analyzing complex biosynthetic pathways. Through the integration of systematic reconstruction, targeted functional interrogation, and efficient heterologous expression, our platform enables rational pathway-level design and facilitates the unlocking of cryptic or silent metabolic potential. By revealing previously inaccessible biosynthetic capabilities, this approach significantly expands the chemical space available for natural product discovery and structural diversification. Beyond its immediate applications, the platform provides a powerful strategy for accelerating advances in drug discovery, optimizing metabolic pathways for industrial biotechnology, and driving innovations at the interface between synthetic biology and natural product engineering.

## Materials and Methods

### Strains, Media, and Growth Conditions

*E. coli* DH10β served as the standard cloning host. Methylation-deficient strains ET12567 and ET12567/pUB307 were utilized for triparental conjugation into *Streptomyces*. *E. coli* strains were cultivated at 37 °C in Luria–Bertani (LB) medium, while *Streptomyces* strains were grown at 30 °C on soy flour mannitol (SFM) plates. Antibiotics were added to the media when necessary, with concentrations as follows: ampicillin at 100 μg/mL, kanamycin at 50 μg/mL, chloramphenicol at 25 μg/mL, apramycin at 50 μg/mL, nalidixic acid at 20 μg/mL. Solid or liquid R5 medium^51^ was employed for investigating actinorhodin production. Intergeneric transfer of plasmids from *E. coli* to *Streptomyces* strains was carried out by triparental conjugation (ET12567/pUB307 × ET12567/*oriT* plasmid × *Streptomyces*) as previously described.^51^

### Golden Gate Domestication and *De Novo* Assembly of the *act* Cluster

The PCR primers for Golden Gate domestication, facilitating the assembly of the *act* cluster using BsaI and PaqCI, were designed with SnapGene Version 6.0.1 (Insightful Science, San Diego, CA). The modular BGC fragment was divided into approximately 2 kb segments with assembly junctions selected from high-fidelity four-base overhang sets. Twelve BGC fragments were subcloned into the pKan plasmid using the NEBuilder HiFi DNA Assembly Master Mix (New England BioLabs, E2621L). The Level 1 GGA reaction included 10 fmol of 4 or 6 subcloned BGC fragments and the pAmp vector. The reaction was performed in a 1× T4 DNA ligase buffer with BsaI-HFv2 (20 units) and T4 DNA ligase (300 units). Similarly, the Level 2 GGA reaction contained 10 fmol of 2 or 3 subcloned Level 1 plasmids and the destination vector pPAP-RFP-BsaI. This reaction was carried out in a 1× T4 DNA ligase buffer with PaqCI (10 units), PaqCI Activator (1 μL), and T4 DNA ligase (300 units). For both levels, the reactions were thermally cycled between 37 and 16 °C for 5 min each over 15 cycles, followed by a heat inactivation step at 60 °C for 5 min. The assembled products were transformed into NEB 10-β electrocompetent cells (New England BioLabs, C3020K) via electroporation, following the manufacturer’s recommended protocol. The assembled plasmid pPAP-*act* was sequenced using Oxford Nanopore Technologes (ONT, Plasmidsaurus).

### Detection and Assay of Actinorhodin in R5 Liquid Medium

For actinorhodin production, 20 μL of spore stock of *Streptomyces* carrying the respective assembled *act* cluster was inoculated into 25 mL of R5 liquid medium. The culture was then grown in a baffled flask placed in a shaking incubator (Innova43, Eppendorf) at 30 °C with a shaking speed of 200 rpm. After 5 days, 1 mL of the culture was mixed with 500 μL of 3N KOH and incubated at 4 °C overnight. The concentration of actinorhodin and γ-actinorhodin combined was assessed by measuring OD_640_ of the supernatant using a spectrophotometer (SmartSpec Plus, Bio-Rad).

### Metabolomics Analysis Using LC-MS/MS

*S. coelicolor* M1152 strains carrying wild-type and mutant *act* clusters were cultured on R5 agar plates. After 4 days of incubation at 30 °C, the agar plates were cut into small pieces and soaked in 25 mL of ethyl acetate. LC-MS/MS acquisition was performed using Shimadzu Nexera X2 UHPLC system, with attached PDA, coupled to Shimadzu 9030QTOF mass spectrometer, equipped with a standard ESI source unit, in which a calibrant delivery system (CDS) is installed. The extracts were injected into a Waters Acquity HSS C18 column (1.8 μm, 100 Å, 2.1 × 100 mm^2^). The column was maintained at 30 °C and run at a flow rate of 0.5 mL/min, using 0.1% formic acid in H_2_O as solvent A, and 0.1% formic acid in acetonitrile as solvent B. A gradient was employed for chromatographic separation starting at 5% B for 1 min, then 5–85% for 9 min, 85–100% for 1 min, and finally held at 100% B for 4 min. The column was re-equilibrated to 5% B for 3 min before the next run was started. All of the samples were analyzed in positive polarity, using data-dependent acquisition mode. In this regard, full scan MS spectra (*m/*z 100–1700, scan rate 10 Hz, ID enabled) were followed by two data-dependent MS/MS spectra (*m*/*z* 100–1700, scan rate 10 Hz, ID disabled) for the two most intense ions per scan. The ions were fragmented using collision-induced dissociation (CID) with fixed collision energy (CE 20 eV) and excluded for 1 s before being reselected for fragmentation. The parameters used for the ESI source were interface voltage 4 kV, interface temperature 300 °C, nebulizing gas flow 3 L/min, and drying gas flow 10 L/min. Samples were randomized before injection, and pooled QC were injected. Raw data obtained from LC-MS analysis were converted to mzXML centroid files by using Shimadzu LabSolutions Postrun Analysis. The files were then imported into MZmine 3 (v3.9.0) for data processing.^52^ For mass detection in positive polarity and when using the algorithm centroid, the noise was set to 1000 for MS1 and 10 for MS2, the option of detecting isotope signals below noise level was selected. For the module, ADAP chromatogram builder to minimum intensity for consecutive scans was set to 3000 and the minimum absolute height to 10,000, using 10 consecutive scans and *m*/*z* tolerance 0.002 *m*/*z* or 10.0 ppm. For peak deconvolution, the local minimum resolver algorithm was used with a chromatographic threshold of 90%, a minimum search range RT of 0.05, a minimum absolute height of 3000, and a minimum ratio peak top/edge of 2.0, peak duration range 0.05–2.5, and minimum scan 10. The ^13^C isotope filter module was used with *m*/*z* tolerance 0.001 *m*/*z* or 5.0 ppm, RT tolerance 0.05, and maximum charge of 3. The isotopic peaks finder module was set using the chemical elements C, H, N, O, and S, and a maximum charge isotope *m*/*z* of 3 and *m*/*z* tolerance of 0.001 *m*/*z* or 0.001 ppm. To align the peak lists, the *m*/*z* tolerance was 0.002 m/z or 10.0 ppm, the weight of *m*/*z* was set at 4, the RT tolerance was 0.1 min, and weight of RT was set at 1. The aligned feature list was filtered using the duplicate filter module with *m*/*z* tolerance 0.001 *m*/*z* or 5.0 ppm, the filter mode new average, and the feature list row filter features present in at least 2 samples. The peak finder module was used, the intensity tolerance was set at 20%, and the RT tolerance at 0.1 min, and *m*/*z* tolerance 00.002 *m*/*z* or 10.0 ppm. In order to build the ion identity network, the correlation group module was used with a minimum feature height of 3000 and an intensity threshold for correlation of 1000. For the ion identity network module, the ion identity library parameters were a maximum charge of 3, maximum molecules per cluster of 3, and adducts [M + H]^+^, [M + Na]^+^, [M + K]^+^, [M + NH_4_]^+^, [M + 2H]^2+^, [M-H + 2Na]^+^, modifications [M-H_2_O], [M-2H_2_O], and [M-3H_2_O]. The resulting feature list was exported to be used for GNPS feature-based molecular networking analysis. The PCA plot was generated using MetaboAnalyst 6.0.^53^

The processed data from MZMine 3 were exported to the Feature-Based Molecular Networking (FBMN) workflow^54^ on GNPS^40^ to build a molecular network. Additionally, MS/MS data were filtered by removing all fragment ions within ±17 Da of the precursor *m*/*z*. A window filter was applied to the MS/MS spectra, retaining only the top six fragment ions within an ±50 Da window across the spectrum. The precursor ion mass and MS/MS fragment ion tolerances were set to 0.01 Da.

For network generation, only edges with cosine similarity values above 0.7 and at least six matched fragment peaks were considered. Furthermore, connections between two nodes were retained only if each was among the other’s top 10 most similar nodes. The maximum molecular family size was limited to 100. MS/MS spectra were matched against GNPS spectral libraries,^55,56^ with library spectra processed using the same filtering criteria as the input data. Retained matches between network and library spectra required a score above 0.7 and at least six matched peaks. Additional edges derived from ion identity network analysis in MZMine 3 were incorporated into the molecular network.^40^ Data were visualized using Cytoscape version 3.10.2.^57^

### Statistical Analysis

Data were analyzed using GraphPad Prism version 9.0 (GraphPad Software, San Diego, CA). The heatmap illustrating the relative abundance of ACT and ACT-related metabolites was generated using MATLAB R2024b (MathWorks, Natick, MA).

## Supplementary Material



## Notes

Map and experimental validation of the assembled ACT BGC (Figures S1 and S2), stop codon scanning of 23 *act* genes (Figures S3 and S4), reannotation of ActI-ORF1 (Figure S5), metabolomic profiling of ACT BGC derivatives (Figures S6–S9), morphology of *Streptomyces* carrying promoter-refactored ACT BGCs (Figure S10); lists of strains, plasmids, sequences, and primers used in this study (Tables S1–S3), annotation of act genes (Table S4), mutations introduced for ACT BGC domestication (Table S5), sequence of synthetic promoter-RBS (Figure S6), comparison of hierarchical GGA with other DNA assembly methods (Table S7) (PDF)

## References

[1] Atanasov A. G., Zotchev S. B., Dirsch V. M., Supuran C. T. (2021). Natural
products in drug discovery: advances and opportunities. Nat. Rev. Drug Discovery.

[2] Broach J. R., Thorner J. (1996). High-throughput screening for drug
discovery. Nature.

[3] Harvey A. L., Edrada-Ebel R., Quinn R. J. (2015). The re-emergence of natural products
for drug discovery in the genomics era. Nat.
Rev. Drug Discovery.

[4] Zerikly M., Challis G. L. (2009). Strategies for the discovery of new natural products
by genome mining. ChemBioChem.

[5] Lewis K., Epstein S., D’Onofrio A., Ling L. L. (2010). Uncultured microorganisms
as a source of secondary metabolites. J. Antibiot..

[6] Musiol-Kroll E. M., Tocchetti A., Sosio M., Stegmann E. (2019). Challenges and advances
in genetic manipulation of filamentous actinomycetes–the remarkable
producers of specialized metabolites. Nat. Prod.
Rep..

[7] Terlouw B.
R., Blin K., Navarro-Muñoz J. C., Avalon N. E., Chevrette M. G., Egbert S., Lee S., Meijer D., Recchia M. J. J., Reitz Z. L., van Santen J. A., Selem-Mojica N., Tørring T., Zaroubi L., Alanjary M., Aleti G., Aguilar C., Al-Salihi S. A. A., Augustijn H. E., Avelar-Rivas J. A., Avitia-Domínguez L. A., Barona-Gómez F., Bernaldo-Agüero J., Bielinski V. A., Biermann F., Booth T. J., Carrion Bravo V. J., Castelo-Branco R., Chagas F. O., Cruz-Morales P., Du C., Duncan K. R., Gavriilidou A., Gayrard D., Gutiérrez-García K., Haslinger K., Helfrich E. J. N., van der Hooft J. J. J., Jati A. P., Kalkreuter E., Kalyvas N., Kang K. B., Kautsar S., Kim W., Kunjapur A. M., Li Y.-X., Lin G.-M., Loureiro C., Louwen J. J. R., Louwen N. L. L., Lund G., Parra J., Philmus B., Pourmohsenin B., Pronk L. J. U., Rego A., Rex D. A. B., Robinson S., Rosas-Becerra L. R., Roxborough E. T., Schorn M. A., Scobie D. J., Singh K. S., Sokolova N., Tang X., Udwary D., Vigneshwari A., Vind K., Vromans S. P. J. M., Waschulin V., Williams S. E., Winter J. M., Witte T. E., Xie H., Yang D., Yu J., Zdouc M., Zhong Z., Collemare J., Linington R. G., Weber T., Medem M. H. (2023). MIBiG 3.0: a community-driven effort to annotate experimentally validated
biosynthetic gene clusters. Nucleic Acids Res..

[8] Blin K., Shaw S., Augustijn H. E., Reitz Z. L., Biermann F., Alanjary M., Fetter A., Terlouw B. R., Metcalf W. W., Helfrich E. J. N., Gilles P., Medema M. H., Weber T. (2023). antiSMASH
7.0: new and improved predictions for detection, regulation, chemical
structures and visualisation. Nucleic Acids
Res..

[9] Van
Bergeijk D. A., Terlouw B. R., Medema M. H., Van Wezel G. P. (2020). Ecology
and genomics of Actinobacteria: new concepts for natural product discovery. Nat. Rev. Microbiol..

[10] Hoskisson P. A., Seipke R. F. (2020). Cryptic or Silent? The Known Unknowns,
Unknown Knowns,
and Unknown Unknowns of Secondary Metabolism. mBio.

[11] Fu J., Bian X., Hu S., Wang H., Huang F., Seibert P. M., Plaza A., Xia L., Müller R., Stewart A. F., Zhang Y. (2012). Full-length RecE enhances linear-linear
homologous recombination and facilitates direct cloning for bioprospecting. Nat. Biotechnol..

[12] Yamanaka K., Reynolds K. A., Kersten R. D., Ryan K. S., Gonzalez D. J., Nizet V., Dorrestein P. C., Moore B. S. (2014). Direct cloning and
refactoring of a silent lipopeptide biosynthetic gene cluster yields
the antibiotic taromycin A. Proc. Natl. Acad.
Sci. U.S.A..

[13] Jiang W., Zhao X., Gabrieli T., Lou C., Ebenstein Y., Zhu T. F. (2015). Cas9-Assisted Targeting of CHromosome segments CATCH
enables one-step targeted cloning of large gene clusters. Nat. Commun..

[14] Enghiad B., Huang C., Guo F., Jiang G., Wang B., Tabatabaei S. K., Martin T. A., Zhao H. (2021). Cas12a-assisted precise
targeted cloning using in vivo Cre-*lox* recombination. Nat. Commun..

[15] Liang M., Liu L., Xu F., Zeng X., Wang R., Yang J., Wang W., Karthik L., Liu J., Yang Z., Zhu G., Wang S., Bai L., Tong Y., Liu X., Wu M., Zhang L.-X., Tan G.-Y. (2022). Activating cryptic biosynthetic gene
cluster through a CRISPR–Cas12a-mediated direct cloning approach. Nucleic Acids Res..

[16] Luo Y., Huang H., Liang J., Wang M., Lu L., Shao Z., Cobb R. E., Zhao H. (2013). Activation and characterization
of a cryptic polycyclic tetramate macrolactam biosynthetic gene cluster. Nat. Commun..

[17] Shao Z., Rao G., Li C., Abil Z., Luo Y., Zhao H. (2013). Refactoring
the silent spectinabilin gene cluster using a plug-and-play scaffold. ACS Synth. Biol..

[18] Kang H.-S., Charlop-Powers Z., Brady S. F. (2016). Multiplexed CRISPR/Cas9-and TAR-mediated
promoter engineering of natural product biosynthetic gene clusters
in yeast. ACS Synth. Biol..

[19] Kim H., Ji C.-H., Je H.-W., Kim J.-P., Kang H.-S. (2020). mpCRISTAR:
multiple plasmid approach for CRISPR/Cas9 and TAR-mediated multiplexed
refactoring of natural product biosynthetic gene clusters. ACS Synth. Biol..

[20] Ji C. H., Kim H., Je H. W., Kwon H., Lee D., Kang H. S. (2022). Top-down
synthetic biology approach for titer improvement of clinically important
antibiotic daptomycin in *Streptomyces roseosporus*. Metab. Eng..

[21] Tong Y. J., Whitford C. M., Robertsen H. L., Blin K., Jorgensen T. S., Klitgaard A. K., Gren T., Jiang X. L., Weber T., Lee S. Y. (2019). Highly
efficient DSB-free base editing for streptomycetes
with CRISPR-BEST. Proc. Natl. Acad. Sci. U.S.A..

[22] Thong W. L., Zhang Y., Zhuo Y., Robins K. J., Fyans J. K., Herbert A. J., Law B. J. C., Micklefield J. (2021). Gene editing
enables rapid engineering of complex antibiotic assembly lines. Nat. Commun..

[23] Nielsen A. A. K., Der B. S., Shin J., Vaidyanathan P., Paralanov V., Strychalski E. A., Ross D., Densmore D., Voigt C. A. (2016). Genetic circuit
design automation. Science.

[24] Zargar A., Lal R., Valencia L., Wang J., Backman T. W. H., Cruz-Morales P., Kothari A., Werts M., Wong A. R., Bailey C. B., Loubat A., Liu Y., Chen Y., Chang S., Benites V. T., Hernández A. C., Barajas J. F., Thompson M. G., Barcelos C., Anayah R., Martin H. G., Mukhopadhyay A., Petzold C. J., Baidoo E. E. K., Katz L., Keasling J. D. (2020). Chemoinformatic-Guided
Engineering of Polyketide Synthases. J. Am.
Chem. Soc..

[25] Podolski, A. ; Lindeboom, T. A. ; Präve, L. ; Kranz, J. ; Schindler, D. ; Bode, H. B. High-throughput engineering and modification of non-ribosomal peptide synthetases based on Golden Gate assembly BioRxiv 2025 2025-04 10.1101/2025.04.23.650154. PMC12668312 41074745

[26] Smanski M. J., Bhatia S., Zhao D., Park Y., Woodruff L. B. A., Giannoukos G., Ciulla D., Busby M., Calderon J., Nicol R. (2014). Functional optimization of gene clusters by combinatorial
design and assembly. Nat. Biotechnol..

[27] Pryor J. M., Potapov V., Bilotti K., Pokhrel N., Lohman G. J. S. (2022). Rapid
40 kb genome construction from 52 parts through data-optimized assembly
design. ACS Synth. Biol..

[28] Bird J. E., Marles-Wright J., Giachino A. (2022). A user’s guide to golden gate
cloning methods and standards. ACS Synth. Biol..

[29] Malpartida F., Hopwood D. A. (1984). Molecular cloning
of the whole biosynthetic pathway
of a *Streptomyces* antibiotic and its expression in
a heterologous host. Nature.

[30] Hashimoto M., Watari S., Taguchi T., Ishikawa K., Kumamoto T., Okamoto S., Ichinose K. (2023). Actinorhodin
biosynthesis terminates
with an unprecedented biaryl coupling reaction. Angew. Chem., Int. Ed..

[31] Potapov V., Ong J. L., Kucera R. B., Langhorst B. W., Bilotti K., Pryor J. M., Cantor E. J., Canton B., Knight T. F., Evans T. C., Lohman G. J. S. (2018). Comprehensive
profiling of four base overhang ligation fidelity by T4 DNA ligase
and application to DNA assembly. ACS Synth.
Biol..

[32] Bai C., van Wezel G. P. (2023). CUBIC:
A versatile cumate-based inducible CRISPRi system
in *Streptomyces*. ACS Synth.
Biol..

[33] Pryor J. M., Potapov V., Kucera R. B., Bilotti K., Cantor E. J., Lohman G. J. S. (2020). Enabling one-pot Golden Gate assemblies of unprecedented
complexity using data-optimized assembly design. PLoS One.

[34] Gomez-Escribano J. P., Bibb M. J. (2011). Engineering *Streptomyces
coelicolor* for heterologous expression of secondary
metabolite gene clusters. Microb. Biotechnol..

[35] Jeong Y., Kim J.-N., Kim M. W., Bucca G., Cho S., Yoon Y. J., Kim B.-G., Roe J.-H., Kim S. C., Smith C. P., Cho B.-K. (2016). The dynamic
transcriptional and translational
landscape of the model antibiotic producer *Streptomyces
coelicolor* A3(2). Nat. Commun..

[36] Rebets Y., Brötz E., Tokovenko B., Luzhetskyy A. (2014). Actinomycetes
biosynthetic potential: how to bridge in silico and in vivo?. J. Ind. Microbiol. Biotechnol..

[37] Taguchi T., Yabe M., Odaki H., Shinozaki M., Metsä-Ketelä M., Arai T., Okamoto S., Ichinose K. (2013). Biosynthetic conclusions from the
functional dissection
of oxygenases for biosynthesis of actinorhodin and related *Streptomyces* antibiotics. Chem. Biol..

[38] Okamoto S., Taguchi T., Ochi K., Ichinose K. (2009). Biosynthesis of actinorhodin
and related antibiotics: discovery of alternative routes for quinone
formation encoded in the *act* gene cluster. Chem. Biol..

[39] Hadfield A. T., Limpkin C., Teartasin W., Simpson T. J., Crosby J., Crump M. P. (2004). The crystal structure
of the *act*III
actinorhodin polyketide reductase: proposed mechanism for ACP and
polyketide binding. Structure.

[40] Wang M., Carver J. J., Phelan V. V., Sanchez L. M., Garg N., Peng Y., Nguyen D. D., Watrous J., Kapono C. A., Luzzatto-Knaan T. (2016). Sharing and community curation of mass spectrometry
data with Global Natural Products Social Molecular Networking. Nat. Biotechnol..

[41] Bai C., Zhang Y., Zhao X., Hu Y., Xiang S., Miao J., Lou C., Zhang L. (2015). Exploiting
a precise
design of universal synthetic modular regulatory elements to unlock
the microbial natural products in *Streptomyces*. Proc. Natl. Acad. Sci. U.S.A..

[42] Lin D., O’Callaghan C. A. (2018). MetClo:
methylase-assisted hierarchical
DNA assembly using a single type IIS restriction enzyme. Nucleic Acids Res..

[43] Hoose A., Vellacott R., Storch M., Freemont P. S., Ryadnov M. G. (2023). DNA synthesis
technologies to close the gene writing gap. Nat. Rev. Chem..

[44] Wu C., Du C., Ichinose K., Choi Y. H., van Wezel G. P. (2017). Discovery
of C-Glycosylpyranonaphthoquinones in *Streptomyces* sp. MBT76 by a Combined NMR-Based Metabolomics and Bioinformatics
Workflow. J. Nat. Prod..

[45] Wu C., Ichinose K., Choi Y. H., van Wezel G. P. (2017). Aromatic
polyketide GTRI-02 is a previously unidentified product of the *act* gene cluster in *Streptomyces coelicolor* A3(2). ChemBioChem.

[46] Hashimoto M., Ishikawa K., Fukushima Y., Shimazu S., Yabuzaki M., Kamezawa Y., Taguchi T., Ichinose K. (2025). Characterization of
ActVI-ORF3 and ActVI-ORF4 as Lactonizing and Delactonizing Enzymes
in Relation to Metabolic Flux in Actinorhodin Biosynthesis. ChemBioChem.

[47] Tong Y., Charusanti P., Zhang L., Weber T., Lee S. Y. (2015). CRISPR-Cas9
based engineering of actinomycetal genomes. ACS Synth. Biol..

[48] Wang G., Zhao Z., Ke J., Engel Y., Shi Y. M., Robinson D., Bingol K., Zhang Z., Bowen B., Louie K., Wang B., Evans R., Miyamoto Y., Cheng K., Kosina S., De Raad M., Silva L., Luhrs A., Lubbe A., Hoyt D. W., Francavilla C., Otani H., Deutsch S., Washton N. M., Rubin E. M., Mouncey N. J., Visel A., Northen T., Cheng J. F., Bode H. B., Yoshikuni Y. (2019). CRAGE enables
rapid activation of
biosynthetic gene clusters in undomesticated bacteria. Nat. Microbiol..

[49] Moore S. J., Lai H.-E., Chee S.-M., Toh M., Coode S., Chengan K., Capel P., Corre C., de los Santos E. L. C., Freemont P. S. (2021). A *Streptomyces venezuelae* Cell-Free Toolkit for Synthetic Biology. ACS
Synth. Biol..

[50] Dietrich L. E. P., Teal T. K., Price-Whelan A., Newman D. K. (2008). Redox-active antibiotics
control gene expression and community behavior in divergent bacteria. Science.

[51] Kieser, T. ; Bibb, M. J. ; Buttner, M. J. ; Chater, K. F. ; Hopwood, D. A. Practical Streptomyces Genetics; John Innes Foundation: Norwich, 2000; Vol. 291.

[52] Schmid R., Heuckeroth S., Korf A., Smirnov A., Myers O., Dyrlund T. S., Bushuiev R., Murray K. J., Hoffmann N., Lu M. (2023). Integrative analysis of multimodal mass spectrometry
data in MZmine 3. Nat. Biotechnol..

[53] Pang Z., Lu Y., Zhou G., Hui F., Xu L., Viau C., Spigelman A. F., MacDonald P. E., Wishart D. S., Li S., Xia J. (2024). MetaboAnalyst 6.0:
towards a unified platform for metabolomics data
processing, analysis and interpretation. Nucleic
Acids Res..

[54] Nothias L.-F., Petras D., Schmid R., Dührkop K., Rainer J., Sarvepalli A., Protsyuk I., Ernst M., Tsugawa H., Fleischauer M. (2020). Feature-based molecular
networking in the GNPS analysis environment. Nat. Methods.

[55] Schmid R., Petras D., Nothias L.-F., Wang M., Aron A. T., Jagels A., Tsugawa H., Rainer J., Garcia-Aloy M., Dührkop K. (2021). Ion identity molecular networking for mass
spectrometry-based metabolomics in the GNPS environment. Nat. Commun..

[56] Wang M., Jarmusch A. K., Vargas F., Aksenov A. A., Gauglitz J. M., Weldon K., Petras D., da Silva R., Quinn R., Melnik A. V. (2020). Mass
spectrometry searches using MASST. Nat. Biotechnol..

[57] Su G., Morris J. H., Demchak B., Bader G. D. (2014). Biological network
exploration with Cytoscape 3. Curr. Protoc.
Bioinf..

