# Supplementary material for: Construction and Diversification of Natural Product Biosynthetic Gene Clusters at High Efficiency and Accuracy
Source: ACS Synth Biol. 2025 Oct 10;14(11):4574–85. doi: 10.1021/acssynbio.5c00601 (PMC12645565; doi:10.1021/acssynbio.5c00601)
Supplement: Supplementary file 1 [file sb5c00601_si_001.pdf]

Supporting infomation for

Construction and diversification of natural product biosynthetic gene clusters at high efficiency and accuracy

Chaoxian Bai*, Lina M. Bayona, Gilles P. van Wezel*

Institute of Biology, Leiden University, Sylviusweg 72, 2333 BE, Leiden, Netherlands.

*Corresponding authors:

Chaoxian Bai. Email: c.bai@biology.leidenuniv.nl; Gilles P. van Wezel. Email: g.wezel@biology.leidenuniv.nl

This PDF file includes:

Supplementary figures S1 to S10
Supplementary tables S1 to S7

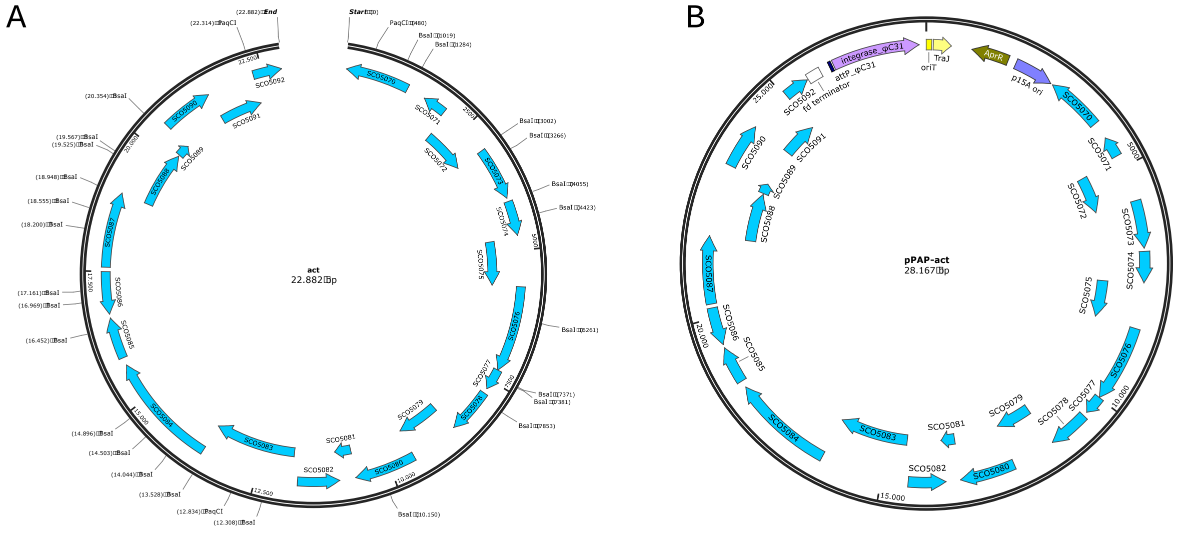

Figure S1. (A) Schematic representation and restriction map of the *act* cluster from *S. coelicolor*, highlighting 25 BsaI and 3 PaqCI restriction sites targeted for removal and subsequent subcloning into the pKan vector. (B) Map of the reassembled *act* cluster in the pPAP vector.

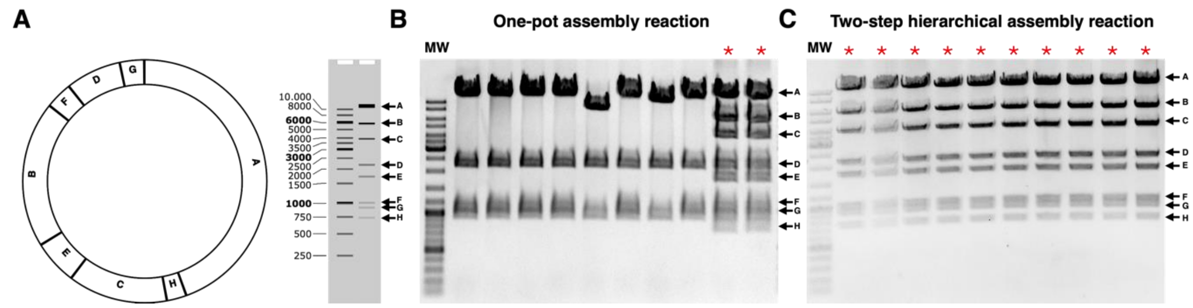

Figure S2. Verification of correct *act* cluster assembly by restriction digestion. (A) Schematic representation of the predicted BamHI digestion pattern of the pPAP-*act* plasmid. Digestion with BamHI yields 8 fragments of the following sizes: A – 11,837 bp; B – 5,804 bp; C – 3,923 bp; D – 2,172 bp; E – 1,731 bp; F – 1,029 bp; G – 928 bp; H – 743 bp. A simulated agarose gel showing the expected BamHI digestion pattern is displayed on the right. (B) Ten colonies randomly selected after transformation with the one-pot assembly reaction were analyzed by BamHI digestion. Only two of the ten clones (indicated by red asterisks) exhibited the correct digestion pattern, indicating successful assembly. (C) Ten colonies randomly selected after transformation with the two-step hierarchical assembly reaction were analyzed by BamHI digestion. All plasmids showed the correct digestion pattern. Red asterisks mark clones with correctly assembled constructs. MW: GeneRuler 1 kb DNA Ladder (Thermo Scientific).

A

B

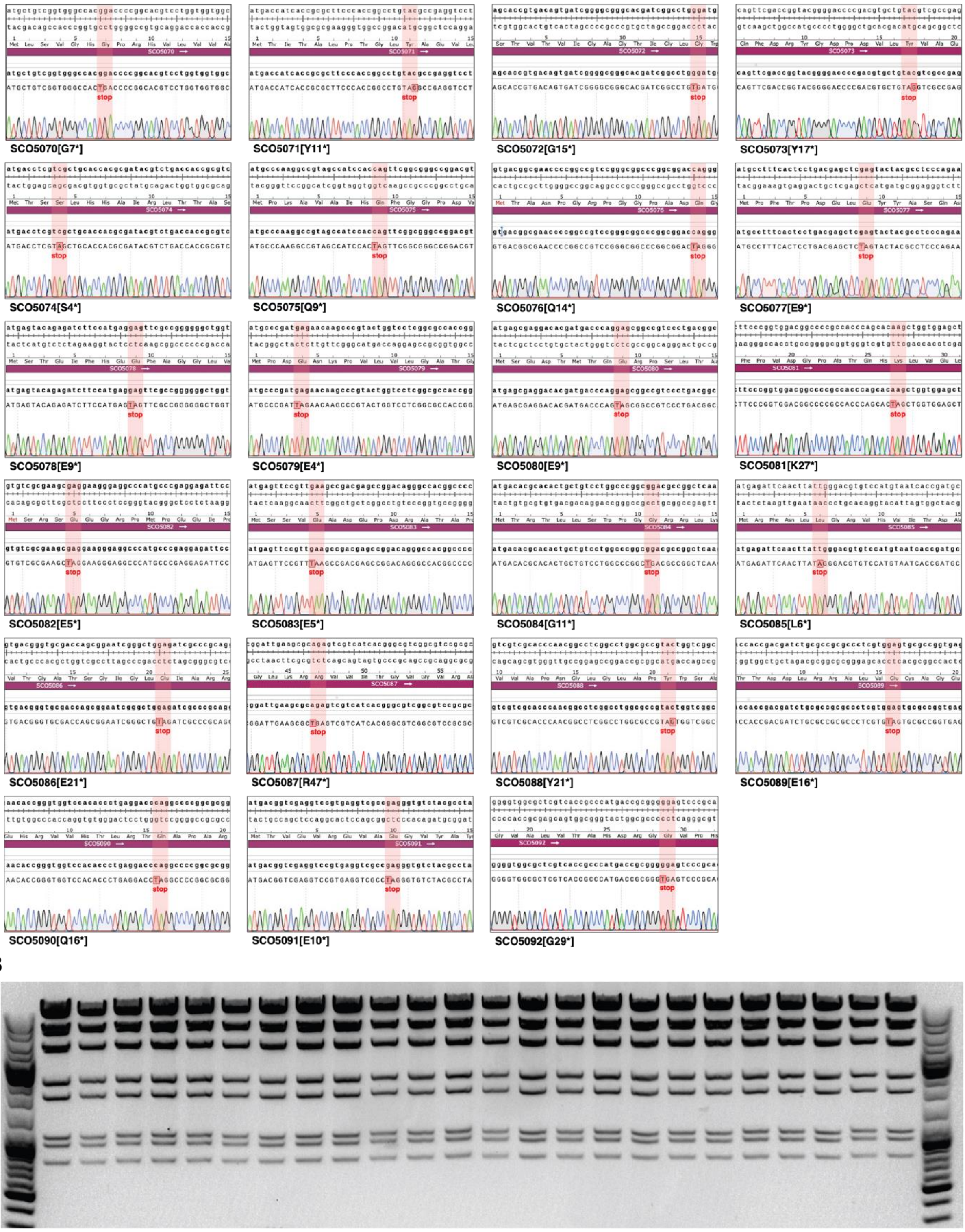

Figure S3. (A) Sanger sequencing traces of 23 genes (SCO5070–5092) within the *act* cluster aligned with the reference sequence. Mutations resulting in the introduction of a stop codon are highlighted in red. (B) BamHI digestion was performed to verify the integrity of the modified BGCs. The digestion pattens are consistent with the results presented in Figure S2.

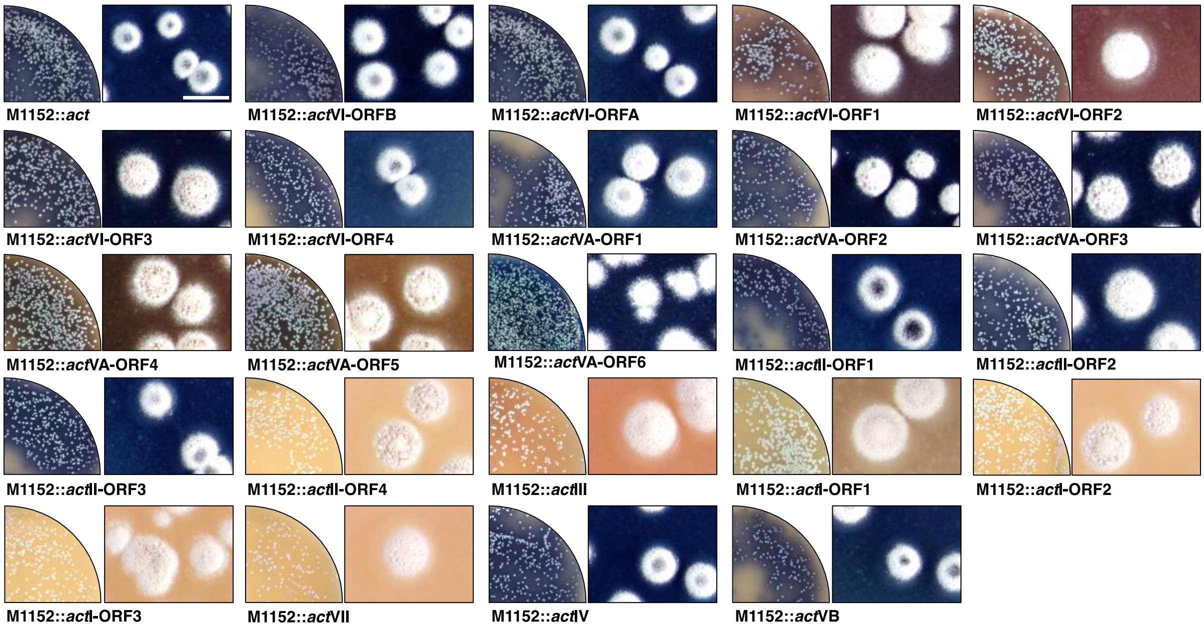

Figure S4. Phenotypes of *S. coelicolor* M1152 ex-conjugants carrying the *act* gene cluster or one of its single-gene mutants grown on SFM agar for 5 days. Bar = 1 mm.

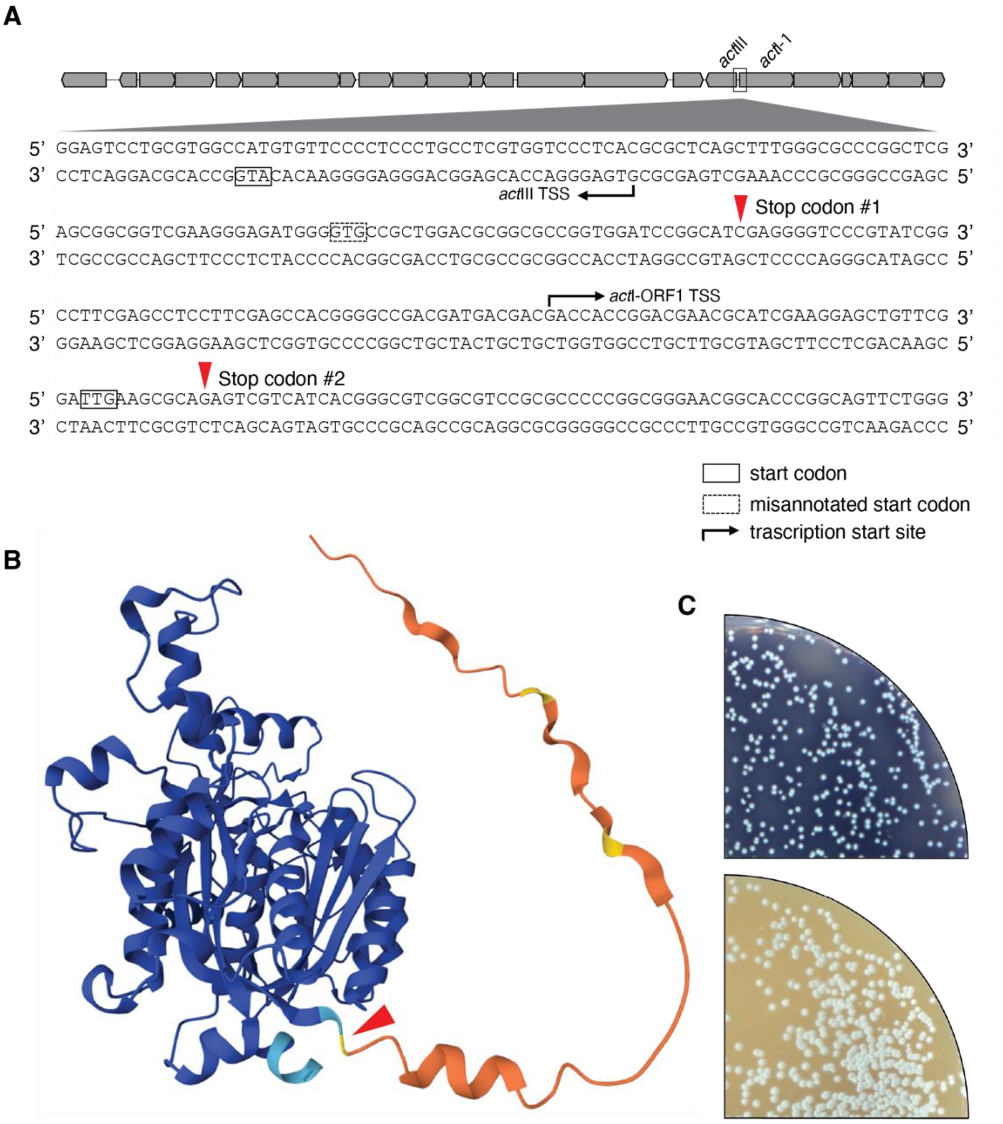

Figure S5. (A) Nucleotide sequences of the *act*III and *act*I-ORF1 promoter regions. Start codons are shown as boxes, and transcription start sites (TSS) are marked with bent arrows. The dashed box highlights the misannotated start codon of *act*I-ORF1. Red arrowheads indicate the two stop codons introduced in *act*I-ORF1, with #1 located upstream of the TSS for *act*I-ORF1. (B) AlphaFold prediction of the annotated ActI-ORF1 reveals that the 43-amino-acid N-terminal extension has very low model confidence. The red arrowhead indicates the correct start codon. (C) Phenotypes of *S. coelicolor* M1152 ex-conjugants carrying the *act*I-ORF1 mutant *act* cluster. Top: Stop codon #1 introduced before the correct start codon — colonies remain blue pigmented. Bottom: Stop codon #2 introduced after the correct start codon — no ACT production observed.

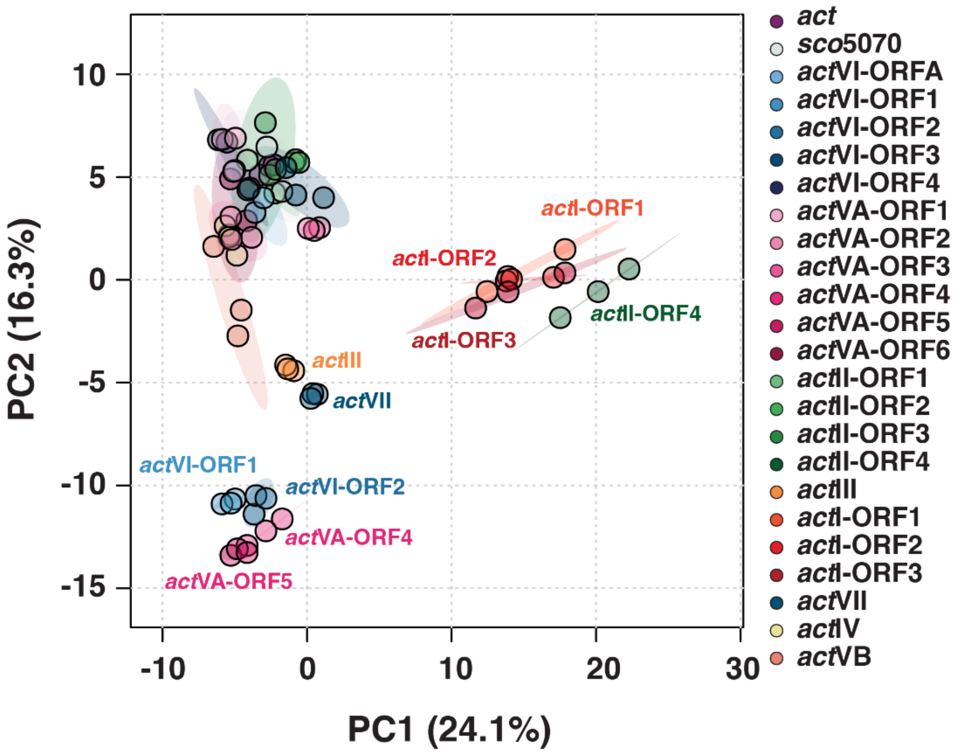

Figure S6. Principal Component Analysis (PCA) score plot of metabolomics data for extracts from the *S. coelicolor* M1152 strains carrying *act* gene cluster and its 23 single-gene mutants grown on R5 agar for 4 days.

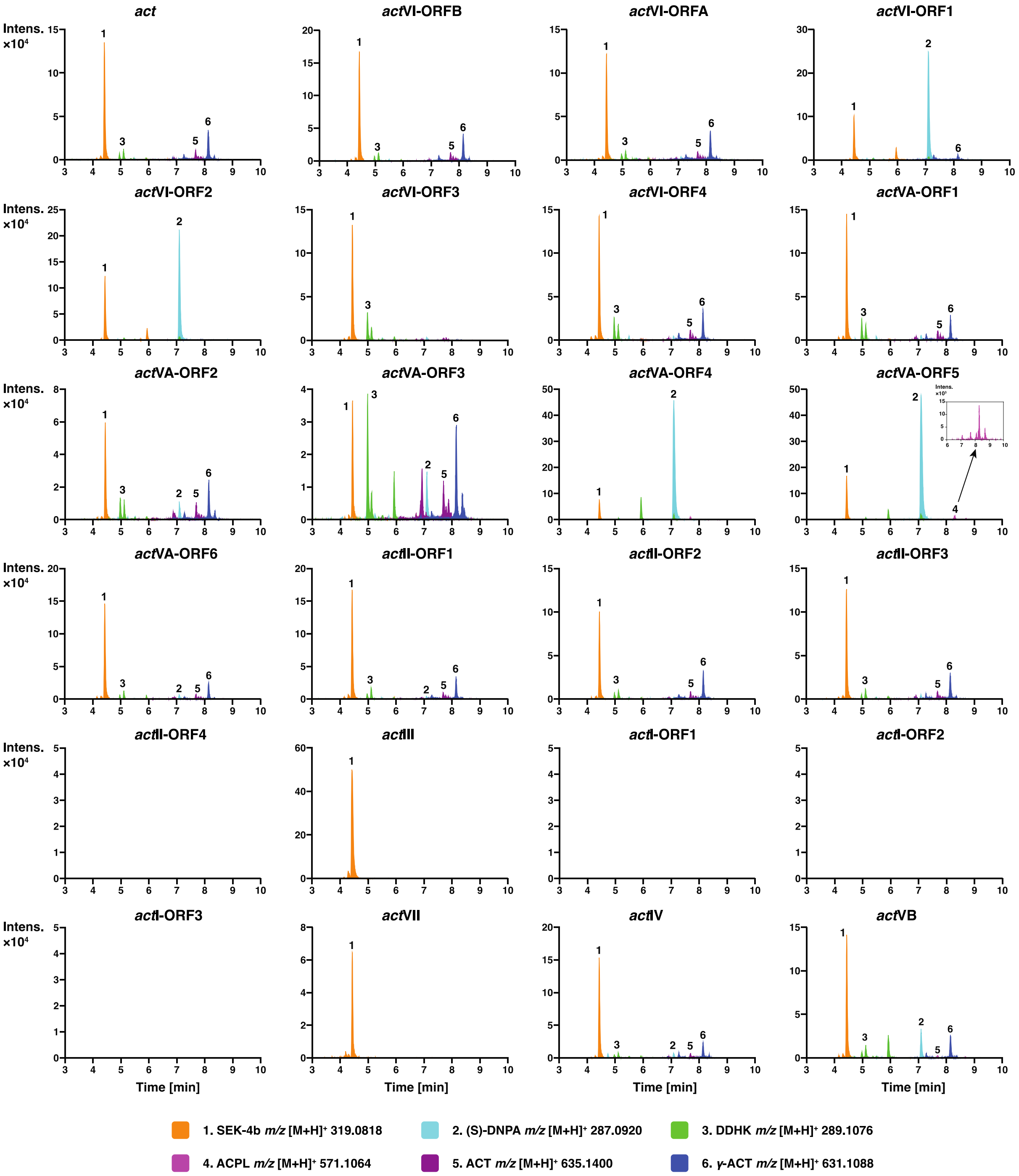

Figure S7. Extracted ion chromatograms (EICs) of actinorhodin (ACT **5** and γ-ACT **6**), its intermediates ((S)-DNPA **2** and DDHK **3**) and shunt products (SEK-4b **1** and ACPL **4**) from extracts of *S. coelicolor* M1152 harboring 24 *act* cluster derivatives grown on R5 agar for 4 days. Data from one representative of three triplicates is shown. The *y*-axis represents intensity of extracted ion chromatograms (EICs) (note: scales are not uniform due to differences in compound abundance across mutant derivatives). A zoom-in of the *act*VA-ORF5 mutant is included to visualize the ACPL peaks.

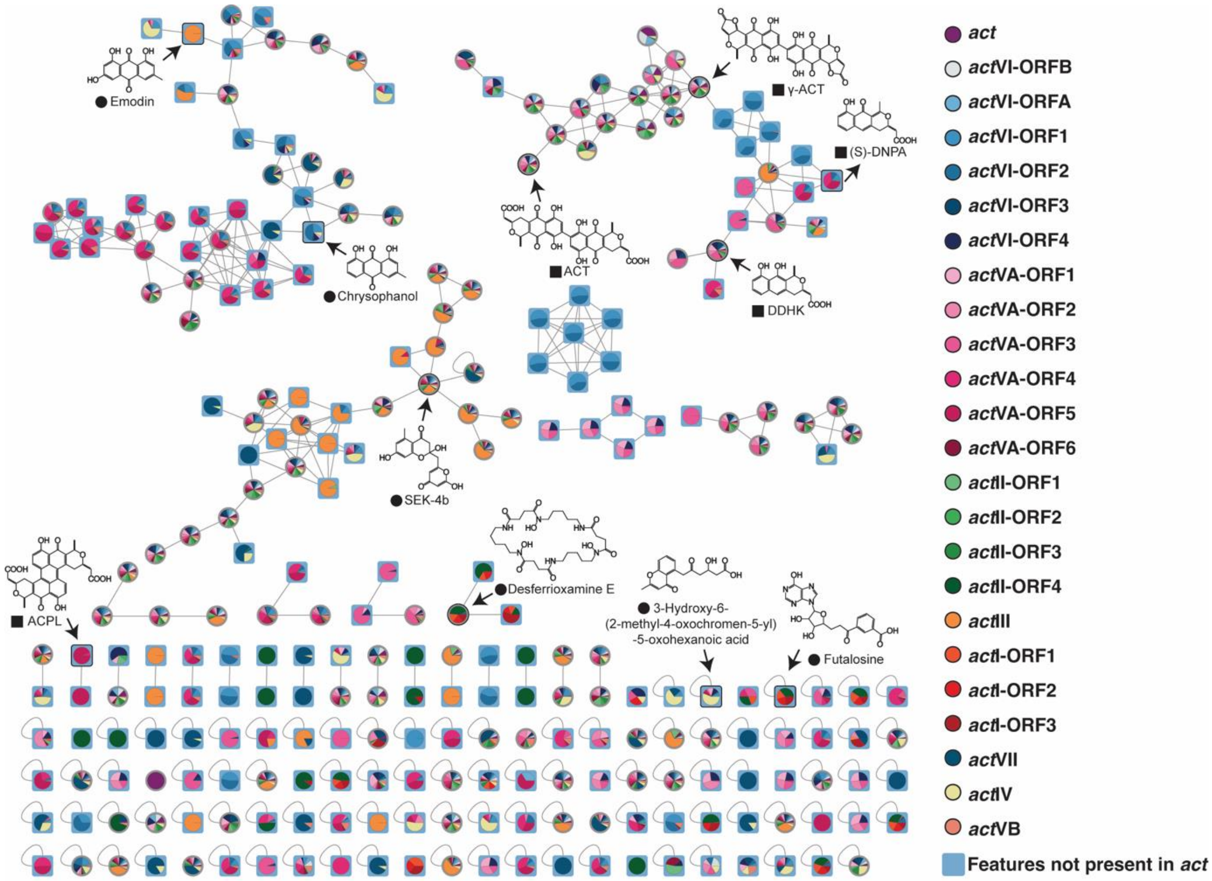

Figure S8. Molecular network of ions detected in crude extracts of *S. coelicolor* M1152 carrying either the wild-type *act* gene cluster or one of its mutant derivatives. Metabolites originating from the chassis strain *S. coelicolor* M1152 were filtered out to focus on ACT-associated compounds. Strains were grown on R5 agar for 4 days prior to preparation of extracts. The relative abundance of compounds is represented by the size of the segments in the pie chart, while different colors indicate the corresponding mutant BGCs. Nodes enclosed in blue squares represent compounds that are absent in the crude extract of *S. coelicolor* M1152 harboring the wild-type *act* gene cluster. Nodes highlighted with their corresponding molecular structures represent compounds annotated either through the GNPS database (●) or via manual identification (■). All matches kept between network spectra and library spectra were required to have a cosine score above 0.7 and at least six matched peaks. Notably, metabolites such as desferrioxamine E and futalosine are not associated with the ACT biosynthetic pathway. Interestingly, these unrelated metabolites are highly enriched in non-producer strains carrying inactive *act* clusters, specifically *act*I-ORF1-3 and *act*II-ORF4 mutants.

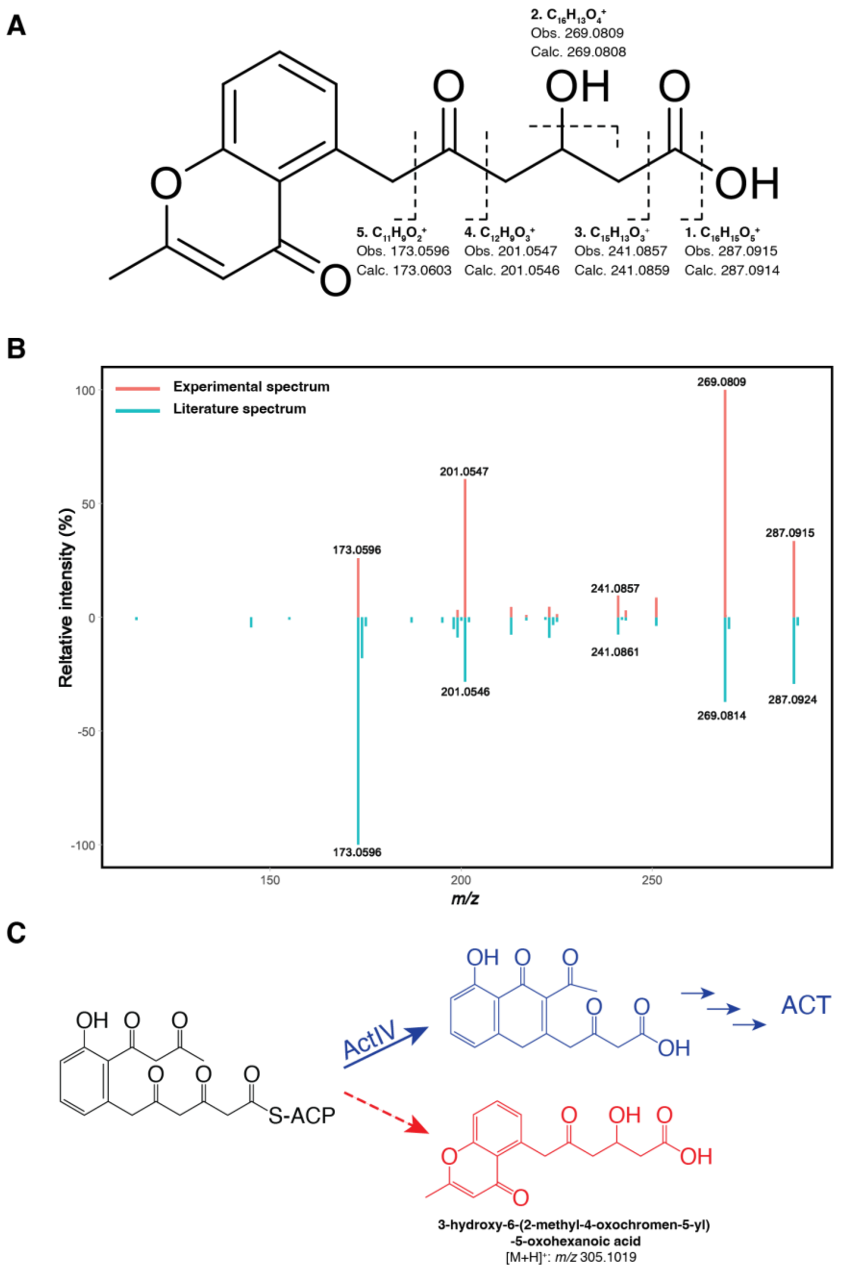

Figure S9. (A) Structure of 3-hydroxy-6-(2-methyl-4-oxochromen-5-yl)-5-oxohexanoic acid, along with its MS/MS fragmentation pattern showing observed and calculated fragment masses. (B) Mirrored MS/MS spectral plot between the experimental MS/MS spectrum (red peaks) and literature MS/MS spectrum (cyan peaks) from the GNPS database. (C) Proposed alternative pathway (shown in red) for second-ring formation in the ActIV mutant.

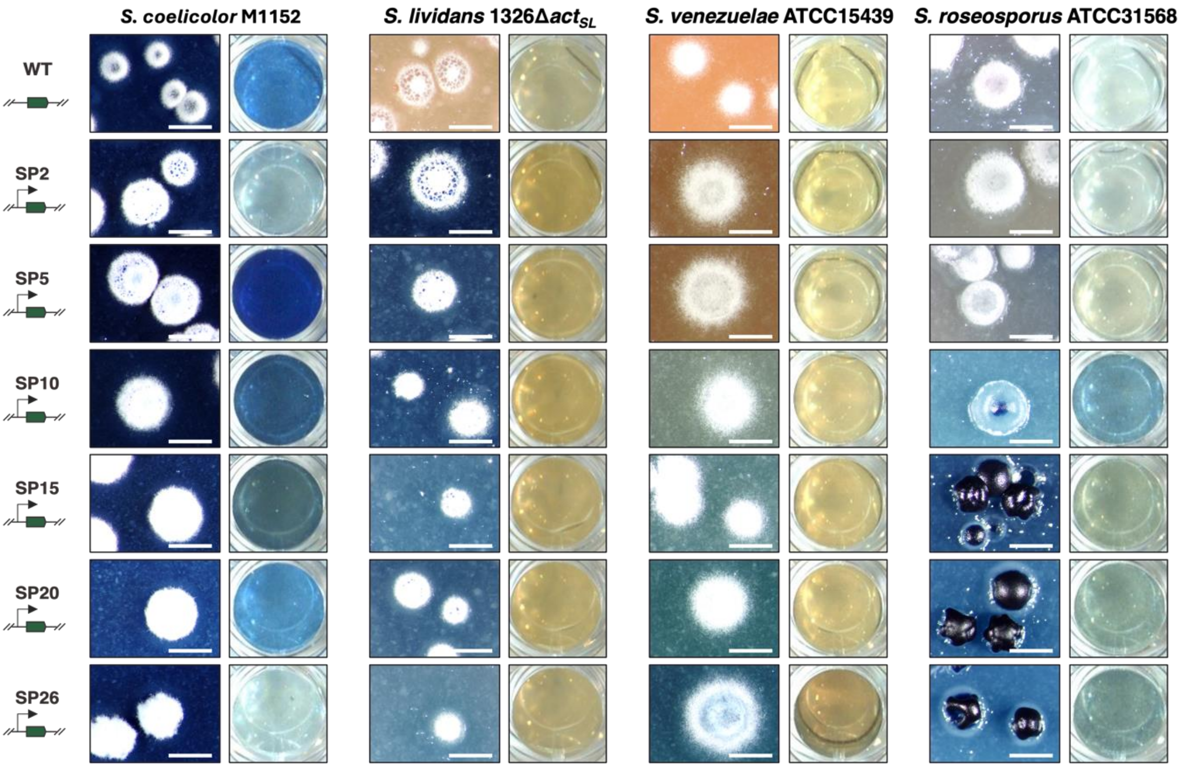

Figure S10. Phenotypes of colonies of *S. coelicolor* M1152, *S. lividans*1326Δ*act*$_{SL}$, *S. venezuelae* ATCC15439 and *S. roseosporus* ATCC31568 carrying either the wild-type or refactored *act* clusters on SFM agar plates. For each species, their corresponding R5 liquid cultures after KOH treatment are displayed on the right column. It is noteworthy that the blue pigmentation produced by *Streptomyces* strains carrying the refactored *act* clusters varies between SFM agar and R5 liquid media. Scale bar, 1 mm.

SUPPLEMENTARY TABLES

Table S1. List of strains used in this study.

Strain	Description	Source
E. coli NEB 10-beta	For routine cloning and plasmid propagation	New England Biolabs
E. coli ET12567	Methylation-deficient host	Maintained in lab
E. coli ET12567/pUB307	ET12567 carrying the self-transmissible plasmid pUB307 for triparental mating	Maintained in lab
S. coelicolor M1152	*S. coelicolor* M145 (Δ*act*, Δ*red*, Δ*cpk*, Δ*cda*, *rpoB* [C2198T])	[1]
S. lividans 1326Δ*act*$_{SL}$	Heterologous *Streptomyces* host for actinorhodin expression, with the native *act* cluster deleted	This study
S. venezuelae ATCC15439	Heterologous *Streptomyces* host for actinorhodin expression	Maintained in lab
S. roseosporus ATCC31568	Heterologous *Streptomyces* host for actinorhodin expression	Maintained in lab
S. coelicolor M1152::*act*	*S. coelicolor* M1152 carrying pPAP-*act*	This study
S. coelicolor M1152::*act*VI-ORFB	*S. coelicolor* M1152 carrying pPAP-*act*-5070	This study
S. coelicolor M1152::*act*VI-ORFA	*S. coelicolor* M1152 carrying pPAP-*act*-5071	This study
S. coelicolor M1152::*act*VI-ORF1	*S. coelicolor* M1152 carrying pPAP-*act*-5072	This study
S. coelicolor M1152::*act*VI-ORF2	*S. coelicolor* M1152 carrying pPAP-*act*-5073	This study
S. coelicolor M1152::*act*VI-ORF3	*S. coelicolor* M1152 carrying pPAP-*act*-5074	This study
S. coelicolor M1152::*act*VI-ORF4	*S. coelicolor* M1152 carrying pPAP-*act*-5075	This study
S. coelicolor M1152::*act*VA-ORF1	*S. coelicolor* M1152 carrying pPAP-*act*-5076	This study
S. coelicolor M1152::*act*VA-ORF2	*S. coelicolor* M1152 carrying pPAP-*act*-5077	This study
S. coelicolor M1152::*act*VA-ORF3	*S. coelicolor* M1152 carrying pPAP-*act*-5078	This study
S. coelicolor M1152::*act*VA-ORF4	*S. coelicolor* M1152 carrying pPAP-*act*-5079	This study
S. coelicolor M1152::*act*VA-ORF5	*S. coelicolor* M1152 carrying pPAP-*act*-5080	This study
S. coelicolor M1152::*act*VA-ORF6	*S. coelicolor* M1152 carrying pPAP-*act*-5081	This study
S. coelicolor M1152::*act*II-ORF1	*S. coelicolor* M1152 carrying pPAP-*act*-5082	This study
S. coelicolor M1152::*act*II-ORF2	*S. coelicolor* M1152 carrying pPAP-*act*-5083	This study
S. coelicolor M1152::*act*II-ORF3	*S. coelicolor* M1152 carrying pPAP-*act*-5084	This study
S. coelicolor M1152::*act*II-ORF4	*S. coelicolor* M1152 carrying pPAP-*act*-5085	This study
S. coelicolor M1152::*act*III	*S. coelicolor* M1152 carrying pPAP-*act*-5086	This study
S. coelicolor M1152::*act*I-ORF1	*S. coelicolor* M1152 carrying pPAP-*act*-5087	This study
S. coelicolor M1152::*act*I-ORF2	*S. coelicolor* M1152 carrying pPAP-*act*-5088	This study

S. coelicolor M1152::*act*I-ORF3	*S. coelicolor* M1152 carrying pPAP-*act*-5089	This study
S. coelicolor M1152::*act*VII	*S. coelicolor* M1152 carrying pPAP-*act*-5090	This study
S. coelicolor M1152::*act*IV	*S. coelicolor* M1152 carrying pPAP-*act*-5091	This study
S. coelicolor M1152::*act*VB	*S. coelicolor* M1152 carrying pPAP-*act*-5092	This study
M1152::*act*-SP2-*act*II-ORF4	*S. coelicolor* M1152 carrying pPAP-*act*-SP2-5085	This study
M1152::*act*-SP5-*act*II-ORF4	*S. coelicolor* M1152 carrying pPAP-*act*-SP5-5085	This study
M1152::*act*-SP10-*act*II-ORF4	*S. coelicolor* M1152 carrying pPAP-*act*-SP10-5085	This study
M1152::*act*-SP15-*act*II-ORF4	*S. coelicolor* M1152 carrying pPAP-*act*-SP15-5085	This study
M1152::*act*-SP20-*act*II-ORF4	*S. coelicolor* M1152 carrying pPAP-*act*-SP20-5085	This study
M1152::*act*-SP26-*act*II-ORF4	*S. coelicolor* M1152 carrying pPAP-*act*-SP26-5085	This study
S. lividans::*act*-SP2-*act*II-ORF4	*S. lividans 1326Δact_{SL}* carrying pPAP-*act*-SP2-5085	This study
S. lividans::*act*-SP5-*act*II-ORF4	*S. lividans 1326Δact_{SL}* carrying pPAP-*act*-SP5-5085	This study
S. lividans:*act*-SP10-*act*II-ORF4	*S. lividans 1326Δact_{SL}* carrying pPAP-*act*-SP10-5085	This study
S. lividans::*act*-SP15-*act*II-ORF4	*S. lividans 1326Δact_{SL}* carrying pPAP-*act*-SP15-5085	This study
S. lividans::*act*-SP20-*act*II-ORF4	*S. lividans 1326Δact_{SL}* carrying pPAP-*act*-SP20-5085	This study
S. lividans::*act*-SP26-*act*II-ORF4	*S. lividans 1326Δact_{SL}* carrying pPAP-*act*-SP26-5085	This study
S. venezuelae::*act*-SP2-*act*II-ORF4	*S. venezuelae* ATCC15439 carrying pPAP-*act*-SP2-5085	This study
S. venezuelae::*act*-SP5-*act*II-ORF4	*S. venezuelae* ATCC15439 carrying pPAP-*act*-SP5-5085	This study
S. venezuelae:*act*-SP10-*act*II-ORF4	*S. venezuelae* ATCC15439 carrying pPAP-*act*-SP10-5085	This study
S. venezuelae::*act*-SP15-*act*II-ORF4	*S. venezuelae* ATCC15439 carrying pPAP-*act*-SP15-5085	This study
S. venezuelae::*act*-SP20-*act*II-ORF4	*S. venezuelae* ATCC15439 carrying pPAP-*act*-SP20-5085	This study
S. venezuelae::*act*-SP26-*act*II-ORF4	*S. venezuelae* ATCC15439 carrying pPAP-*act*-SP26-5085	This study
S. roseosporus::*act*-SP2-*act*II-ORF4	*S. roseosporus* ATCC31568 carrying pPAP-*act*-SP2-5085	This study
S. roseosporus::*act*-SP5-*act*II-ORF4	*S. roseosporus* ATCC31568 carrying pPAP-*act*-SP5-5085	This study
S. roseosporus:*act*-SP10-*act*II-ORF4	*S. roseosporus* ATCC31568 carrying pPAP-*act*-SP10-5085	This study
S. roseosporus::*act*-SP15-*act*II-ORF4	*S. roseosporus* ATCC31568 carrying pPAP-*act*-SP15-5085	This study
S. roseosporus::*act*-SP20-*act*II-ORF4	*S. roseosporus* ATCC31568 carrying pPAP-*act*-SP20-5085	This study
S. roseosporus::*act*-SP26-*act*II-ORF4	*S. roseosporus* ATCC31568 carrying pPAP-*act*-SP26-5085	This study

Tables S2. List of plasmids used in this study.

Plasmid	Description
pKan	Entry vector for subcloning BGC fragments, pUC origin, KanR
pAmp2.1	Level 1 vector for assembly 6 BGC fragments with overhangs TCTG (5'to 3') and TCAA (5'to 3'), pUC origin, Amp^R
pAmp2.2	Level 1 vector for assembly 6 BGC fragments with overhangs TTGA (5'to 3') and ACTA (5'to 3'), pUC origin, Amp^R
pAmp3.1	Level 1 vector for assembly 4 BGC fragments with overhangs TCTG (5'to 3') and ATAC (5'to 3'), pUC origin, Amp^R
pAmp3.2	Level 1 vector for assembly 4 BGC fragments with overhangs GTAT (5'to 3') and TGAA (5'to 3'), pUC origin, Amp^R
pAmp3.3	Level 1 vector for assembly 4 BGC fragments with overhangs TTCA (5'to 3') and ACTA (5'to 3'), pUC origin, Amp^R
pPAP-BsaI	Level 2 vector for assembly full BGC from 12 subcloned BGC fragments, p15A origin, Apr^R, φC31 integrase, *oriT-traJ*
pPAP-PaqCI	Level 2 vector for assembly full BGC from 2 or 3 level 1 BGC assemblies, p15A origin, Apr^R, φC31 integrase, *oriT-traJ*
pKan-*act*12.1	Fragment #1 of *act* cluster in pKan
pKan-*act*12.2	Fragment #2 of *act* cluster in pKan
pKan-*act*12.3	Fragment #3 of *act* cluster in pKan
pKan-*act*12.4	Fragment #4 of *act* cluster in pKan
pKan-*act*12.5	Fragment #5 of *act* cluster in pKan
pKan-*act*12.6	Fragment #6 of *act* cluster in pKan
pKan-*act*12.7	Fragment #7 of *act* cluster in pKan
pKan-*act*12.8	Fragment #8 of *act* cluster in pKan
pKan-*act*12.9	Fragment #9 of *act* cluster in pKan
pKan-*act*12.10	Fragment #10 of *act* cluster in pKan
pKan-*act*12.11	Fragment #11 of *act* cluster in pKan
pKan-*act*12.12	Fragment #12 of *act* cluster in pKan
pAmp-*act*2.1	Fragments from #1 to #6 of *act* cluster in pAmp2.1
pAmp-*act*2.2	Fragments from #7 to #12 of *act* cluster in pAmp2.2
pAmp-*act*3.1	Fragments from #1 to #4 of *act* cluster in pAmp3.1
pAmp-*act*3.2	Fragments from #5 to #8 of *act* cluster in pAmp3.2
pAmp-*act*3.3	Fragments from #9 to #12 of *act* cluster in pAmp3.3
pPAP-*act*	Full *act* cluster assembled in pPAP
pKan-*act*12.8-SP2	SP2 promoter upstream of *act*II-ORF4 in pKan-*act*12.8
pKan-*act*12.8-SP5	SP5 promoter upstream of *act*II-ORF4 in pKan-*act*12.8
pKan-*act*12.8-SP10	SP10 promoter upstream of *act*II-ORF4 in pKan-*act*12.8
pKan-*act*12.8-SP15	SP15 promoter upstream of *act*II-ORF4 in pKan-*act*12.8
pKan-*act*12.8-SP20	SP20 promoter upstream of *act*II-ORF4 in pKan-*act*12.8
pKan-*act*12.8-SP26	SP26 promoter upstream of *act*II-ORF4 in pKan-*act*12.8
pKan-*act*12.1-5070	Stop codon introduced into *sco*5070 in pKan-*act*12.1
pKan-*act*12.1-5071	Stop codon introduced into *sco*5071 in pKan-*act*12.1
pKan-*act*12.2-5072	Stop codon introduced into *sco*5072 in pKan-*act*12.2
pKan-*act*12.2-5073	Stop codon introduced into *sco*5073 in pKan-*act*12.2
pKan-*act*12.3-5074	Stop codon introduced into *sco*5074 in pKan-*act*12.3
pKan-*act*12.3-5075	Stop codon introduced into *sco*5075 in pKan-*act*12.3
pKan-*act*12.3-5076	Stop codon introduced into *sco*5076 in pKan-*act*12.3
pKan-*act*12.4-5077	Stop codon introduced into *sco*5077 in pKan-*act*12.4
pKan-*act*12.4-5078	Stop codon introduced into *sco*5078 in pKan-*act*12.4

pKan-*act*12.5-5079	Stop codon introduced into *sco*5079 in pKan-*act*12.5
pKan-*act*12.5-5080	Stop codon introduced into *sco*5080 in pKan-*act*12.5
pKan-*act*12.6-5081	Stop codon introduced into *sco*5081 in pKan-*act*12.6
pKan-*act*12.6-5082	Stop codon introduced into *sco*5082 in pKan-*act*12.6
pKan-*act*12.6-5083	Stop codon introduced into *sco*5083 in pKan-*act*12.6
pKan-*act*12.7-5084	Stop codon introduced into *sco*5084 in pKan-*act*12.7
pKan-*act*12.8-5085	Stop codon introduced into *sco*5085 in pKan-*act*12.8
pKan-*act*12.9-5086	Stop codon introduced into *sco*5086 in pKan-*act*12.9
pKan-*act*12.10-5087	Stop codon introduced into *sco*5087 in pKan-*act*12.10
pKan-*act*12.10-5088	Stop codon introduced into *sco*5088 in pKan-*act*12.10
pKan-*act*12.11-5089	Stop codon introduced into *sco*5089 in pKan-*act*12.11
pKan-*act*12.11-5090	Stop codon introduced into *sco*5090 in pKan-*act*12.11
pKan-*act*12.12-5091	Stop codon introduced into *sco*5091 in pKan-*act*12.12
pKan-*act*12.12-5092	Stop codon introduced into *sco*5092 in pKan-*act*12.12
pAmp-*act*3.1-5070	Fragments from #1 to #4 of *act* cluster in pAmp3.1 with stop codon introduced in *sco*5070
pAmp-*act*3.1-5071	Fragments from #1 to #4 of *act* cluster in pAmp3.1 with stop codon introduced in *sco*5071
pAmp-*act*3.1-5072	Fragments from #1 to #4 of *act* cluster in pAmp3.1 with stop codon introduced in *sco*5072
pAmp-*act*3.1-5073	Fragments from #1 to #4 of *act* cluster in pAmp3.1 with stop codon introduced in *sco*5073
pAmp-*act*3.1-5074	Fragments from #1 to #4 of *act* cluster in pAmp3.1 with stop codon introduced in *sco*5074
pAmp-*act*3.1-5075	Fragments from #1 to #4 of *act* cluster in pAmp3.1 with stop codon introduced in *sco*5075
pAmp-*act*3.1-5076	Fragments from #1 to #4 of *act* cluster in pAmp3.1 with stop codon introduced in *sco*5076
pAmp-*act*3.1-5077	Fragments from #1 to #4 of *act* cluster in pAmp3.1 with stop codon introduced in *sco*5077
pAmp-*act*3.1-5078	Fragments from #1 to #4 of *act* cluster in pAmp3.1 with stop codon introduced in *sco*5078
pAmp-*act*3.2-5079	Fragments from #5 to #8 of *act* cluster in pAmp3.2 with stop codon introduced in *sco*5079
pAmp-*act*3.2-5080	Fragments from #5 to #8 of *act* cluster in pAmp3.2 with stop codon introduced in *sco*5080
pAmp-*act*3.2-5081	Fragments from #5 to #8 of *act* cluster in pAmp3.2 with stop codon introduced in *sco*5081
pAmp-*act*3.2-5082	Fragments from #5 to #8 of *act* cluster in pAmp3.2 with stop codon introduced in *sco*5082
pAmp-*act*3.2-5083	Fragments from #5 to #8 of *act* cluster in pAmp3.2 with stop codon introduced in *sco*5083
pAmp-*act*3.2-5084	Fragments from #5 to #8 of *act* cluster in pAmp3.2 with stop codon introduced in *sco*5084
pAmp-*act*3.2-5085	Fragments from #5 to #8 of *act* cluster in pAmp3.2 with stop codon introduced in *sco*5085
pAmp-*act*3.3-5086	Fragments from #9 to #12 of *act* cluster in pAmp3.3 with stop codon introduced in *sco*5086
pAmp-*act*3.3-5087	Fragments from #9 to #12 of *act* cluster in pAmp3.3 with stop codon introduced in *sco*5087
pAmp-*act*3.3-5088	Fragments from #9 to #12 of *act* cluster in pAmp3.3 with stop codon introduced in *sco*5088
pAmp-*act*3.3-5089	Fragments from #9 to #12 of *act* cluster in pAmp3.3 with stop codon introduced in *sco*5089

pAmp-*act*3.3-5090	Fragments from #9 to #12 of *act* cluster in pAmp3.3 with stop codon introduced in *sco*5090
pAmp-*act*3.3-5091	Fragments from #9 to #12 of *act* cluster in pAmp3.3 with stop codon introduced in *sco*5091
pAmp-*act*3.3-5092	Fragments from #9 to #12 of *act* cluster in pAmp3.3 with stop codon introduced in *sco*5092
pAmp-*act*3.2-SP2-5085	Fragments from #5 to #8 of *act* cluster in pAmp3.2 with *act*II-ORF4 promoter replaced by SP2
pAmp-*act*3.2-SP5-5085	Fragments from #5 to #8 of *act* cluster in pAmp3.2 with *act*II-ORF4 promoter replaced by SP5
pAmp-*act*3.2-SP10-5085	Fragments from #5 to #8 of *act* cluster in pAmp3.2 with *act*II-ORF4 promoter replaced by SP10
pAmp-*act*3.2-SP15-5085	Fragments from #5 to #8 of *act* cluster in pAmp3.2 with *act*II-ORF4 promoter replaced by SP15
pAmp-*act*3.2-SP20-5085	Fragments from #5 to #8 of *act* cluster in pAmp3.2 with *act*II-ORF4 promoter replaced by SP20
pAmp-*act*3.2-SP26-5085	Fragments from #5 to #8 of *act* cluster in pAmp3.2 with *act*II-ORF4 promoter replaced by SP26
pPAP-*act*-SP2-5085	Full *act* cluster assembled in pPAP with *act*II-ORF4 promoter replaced by SP2
pPAP-*act*-SP5-5085	Full *act* cluster assembled in pPAP with *act*II-ORF4 promoter replaced by SP5
pPAP-*act*-SP10-5085	Full *act* cluster assembled in pPAP with *act*II-ORF4 promoter replaced by SP10
pPAP-*act*-SP15-5085	Full *act* cluster assembled in pPAP with *act*II-ORF4 promoter replaced by SP15
pPAP-*act*-SP20-5085	Full *act* cluster assembled in pPAP with *act*II-ORF4 promoter replaced by SP20
pPAP-*act*-SP26-5085	Full *act* cluster assembled in pPAP with *act*II-ORF4 promoter replaced by SP26
pPAP-*act*-5070	Full *act* cluster assembled in pPAP with stop codon introduced in *sco*5070
pPAP-*act*-5071	Full *act* cluster assembled in pPAP with stop codon introduced in *sco*5071
pPAP-*act*-5072	Full *act* cluster assembled in pPAP with stop codon introduced in *sco*5072
pPAP-*act*-5073	Full *act* cluster assembled in pPAP with stop codon introduced in *sco*5073
pPAP-*act*-5074	Full *act* cluster assembled in pPAP with stop codon introduced in *sco*5074
pPAP-*act*-5075	Full *act* cluster assembled in pPAP with stop codon introduced in *sco*5075
pPAP-*act*-5076	Full *act* cluster assembled in pPAP with stop codon introduced in *sco*5076
pPAP-*act*-5077	Full *act* cluster assembled in pPAP with stop codon introduced in *sco*5077
pPAP-*act*-5078	Full *act* cluster assembled in pPAP with stop codon introduced in *sco*5078
pPAP-*act*-5079	Full *act* cluster assembled in pPAP with stop codon introduced in *sco*5079
pPAP-*act*-5080	Full *act* cluster assembled in pPAP with stop codon introduced in *sco*5080
pPAP-*act*-5081	Full *act* cluster assembled in pPAP with stop codon introduced in *sco*5081
pPAP-*act*-5082	Full *act* cluster assembled in pPAP with stop codon introduced in *sco*5082
pPAP-*act*-5083	Full *act* cluster assembled in pPAP with stop codon introduced in *sco*5083
pPAP-*act*-5084	Full *act* cluster assembled in pPAP with stop codon introduced in *sco*5084
pPAP-*act*-5085	Full *act* cluster assembled in pPAP with stop codon introduced in *sco*5085
pPAP-*act*-5086	Full *act* cluster assembled in pPAP with stop codon introduced in *sco*5086
pPAP-*act*-5087	Full *act* cluster assembled in pPAP with stop codon introduced in *sco*5087
pPAP-*act*-5088	Full *act* cluster assembled in pPAP with stop codon introduced in *sco*5088
pPAP-*act*-5089	Full *act* cluster assembled in pPAP with stop codon introduced in *sco*5089
pPAP-*act*-5090	Full *act* cluster assembled in pPAP with stop codon introduced in *sco*5090
pPAP-*act*-5091	Full *act* cluster assembled in pPAP with stop codon introduced in *sco*5091
pPAP-*act*-5092	Full *act* cluster assembled in pPAP with stop codon introduced in *sco*5092

Table S3. Primers used in this study.

Name	Sequence (5' to 3')
***act* gene cluster domestication**	
ACT-P1-1F	gcaacgcggccttggtctcacagactcacggtcggccgatcccc
ACT-P1-1R	accgccgtgttcgagagggc
ACT-P1-2F	ctctcgaacacggcggtgacctgccggccgccctcc
ACT-P1-2R	gacggccggccccgccccgt
ACT-P1-3F	acggggcggggccggccgtcgccgtctcgatgccctcgctcag
ACT-P1-3R	gtcggcgcgacggctggtctgccgtttcccgggcaccggcc
ACT-P1-4F	agaccagccgtcgcgccgac
ACT-P1-4R	cgcgcacatttccggtctcagctcgagggaacttcgaggc
ACT-P2-1F	gcaacgcggccttggtctcagagccgggttcgagtgggcg
ACT-P2-1R	ctgcacggcacgcatcacttcgtctcctcctggggctgctc
ACT-P2-2F	aagtgatgcgtgccgtgcag
ACT-P2-2R	gtgaacgcaccgacccggtcgcccggccgcagggccgtgac
ACT-P2-3F	gaccgggtcggtgcgttcac
ACT-P2-3R	cgcgcacatttccggtctccttcgggctcttgg
ACT-P3-1F	gcaacgcggccttggtctcacgaaggagacgtttccatgacctcgtcgctg
ACT-P3-1R	cggacgtcggagtcgccgaggccgctgccgccggtgagccg
ACT-P3-2F	ctcggcgactccgacgtccg
ACT-P3-2R	cgcgcacatttccggtctcgggcaccaccagca
ACT-P4-1F	gcaacgcggccttggtctcatgcccgagacgcggggcacggcgggcaggcg
ACT-P4-1R	tctcggtgttgacgcggaacgacaccgggttggcctggggctg
ACT-P4-2F	gttccgcgtcaacaccgagacgggcacgatcgacatcggcgg
ACT-P4-2R	agcccgtctcccgcctcgaggccgtcgaccgggcgaagccg
ACT-P4-3F	ctcgaggcgggagacgggct
ACT-P4-3R	cgcgcacatttccggtctcagtatccgacctcgagccggt
ACT-P5-1F	gcaacgcggccttggtctcaatacgacgtcgcggccggca
ACT-P5-1R	cctgccgcgatggccgcacg
ACT-P5-2F	cgtgcggccatcgcggcaggcctcggtccggatgccgaggc
ACT-P5-2R	cgcgcacatttccggtctcaatctcacccgtggcccgggc
ACT-P6-1F	gcaacgcggccttggtctcaagatcgacgcggcccagctg
ACT-P6-1R	cgcgcacatttccggtctcattgatgaggccgagcccttgcggaatcat
ACT-P7-1F	gcaacgcggccttggtctcatcaagcaaatgtt
ACT-P7-1R	ggtgcagggcgagcagcagc
ACT-P7-2F	ctgctcgccctgcaccggcagatcggcctgggctt
ACT-P7-2R	accgccagcagtgcgatcac
ACT-P7-3F	gtgatcgcactgctggcggtgtccttcgcgctggccttccg
ACT-P7-3R	ccgtccggggcgctcggccg
ACT-P7-4F	cggccgagcgccccggacggcctcggcgtccacgtcacggg
ACT-P7-4R	cgcgcacatttccggtctcaccttggtggagttcagagcg
ACT-P8-1F	gcaacgcggccttggtctcaaagggcctcggcccggtctgcgccgt
ACT-P8-1R	acctcgatgacgccgggcaccgtctccagagctgcgtgcac
ACT-P8-2F	gtgcccggcgtcatcgaggt
ACT-P8-2R	cgcgcacatttccggtctcgttcagcggatgcg
ACT-P9-1F	gcaacgcggccttggtctcatgaacgagacgctgcacgccaaactgatggg
ACT-P9-1R	ctcgaactggcgaggaccgg
ACT-P9-2F	ccggtcctcgccagttcgaggccgagcgccttggtgaagcc

ACT-P9-2R	aacctcaccggcgtgttccg
ACT-P9-3F	cggaacacgccggtgaggttcgtctccacgacatcgagcca
ACT-P9-3R	cgcgcacatttccggtctcagctgagcgcgtgagggacc
ACT-P10-1F	gcaacgcggccttggtctcacagctttgggcgcccggctc
ACT-P10-1R	accatcgtgaccgggccctc
ACT-P10-2F	gagggcccggtcacgatggtgtccaccggctgcacctcggg
ACT-P10-2R	gtctcggccatctcgcggcc
ACT-P10-3F	ggccgcgagatggccgagacgatccgggtcgccctcgacga
ACT-P10-3R	gtctcggcgtcgcgcagcac
ACT-P10-4F	gtgctgcgcgacgccgagacggcgggggcggccgcatgagc
ACT-P10-4R	cgcgcacatttccggtctcagaagtcgaacccaccgcagg
ACT-P11-1F	gcaacgcggccttggtctcacttcacccaccgggagttc
ACT-P11-1R	acgggtcgagtgccgagtccaccccgccggacaccaccagcggcgtgccgcg
ACT-P11-2F	ggactcggcactcgacccgtggggctgggtgtcgcagatcgccagtggccg
ACT-P11-2R	gtctccatcagggcgagcga
ACT-P11-3F	tcgctcgccctgatggagacggcggcgcgactcgagagccg
ACT-P11-3R	cgcgcacatttccggtctcatcaccgtctccatggtcatg
ACT-P12-1F	gcaacgcggccttggtctcagtgaccaccgacggcggtac
ACT-P12-1R	ggcagctgcccaccgttgta
ACT-P12-2F	acggtgggcagctgccgacctgcctcgcctaggga
ACT-P12-2R	cgcgcacatttccggtctcatagtcggccttcccggccggcctc
**Stop codon scanning mutagenesis**	
SCO5070-stop-F	agtggcccaccgacagcatc
SCO5070-stop-R	gatgctgtcggtgggccactgaccccggcacgtcctggtg
SCO5071-stop-F	ctacaggccggtgggaagcg
SCO5071-stop-R	cgcttcccaccggcctgtaggccgaggtcctgtcgttcta
SCO5072-stop-F	tgatggatcaacctgttcag
SCO5072-stop-R	ctgaacaggttgatccatcacaggccgatcgtgcccgccc
SCO5073-stop-F	ggtcgccgagcgtcccgtcc
SCO5073-stop-R	ggacgggacgctcggcgacctacagcacgtcggggtcccc
SCO5074-stop-F	agctgcaccacgcgatacgt
SCO5074-stop-R	acgtatcgcgtggtgcagctacgaggtcatggaaacgtct
SCO5075-stop-F	tagttcggcgggccggacgt
SCO5075-stop-R	acgtccggcccgccgaactagtggatggctacggccttgg
SCO5076-stop-F	tagggccacccgcgccgctg
SCO5076-stop-R	cagcggcgcgggtggccctagtccgccgggccgcccggac
SCO5077-stop-F	tagtactacgcctcccagaa
SCO5077-stop-R	ttctgggaggcgtagtactagagctcgtcaggagtgaaag
SCO5078-stop-F	tagttcgccggggggctggt
SCO5078-stop-R	accagccccccggcgaactactcatggaagatctctgtac
SCO5079-stop-F	tagaacaagcccgtactggt
SCO5079-stop-R	accagtacgggcttgttctaatcgggcatcgcagtgcctt
SCO5080-stop-F	tagcggccgtccctgacggc
SCO5080-stop-R	gccgtcagggacggccgctactgggtcatcgtgtcctcgc
SCO5081-stop-F	tagctggtggagctggccacc
SCO5081-stop-R	ggtggccagctccaccagctagtgctgggtggcggggccgt
SCO5082-stop-F	gcttcgcgacacgtgctcct

SCO5082-stop-R	ggagcacgtgtcgcgaagctaggaagggaggcccatgccc
SCO5083-stop-F	aagccgacgagccggacagg
SCO5083-stop-R	ctgtccggctcgtcggcttaaacggaactcatggcgtccc
SCO5084-stop-F	tgacgccggctcaagtggct
SCO5084-stop-R	agccacttgagccggcgtcagccgggccaggacagcagtg
SCO5085-stop-F	ggacacgtccctataagttgaatctcatctgcgc
SCO5085-stop-R	caacttatagggacgtgtccatgtaatcac
SCO5086-stop-F	acagcccgattccgctggtc
SCO5086-stop-R	gaccagcggaatcgggctgtagatcgcccgcaggctcggt
SCO5087-stop-F	agaggggtcccgtatcggcc
SCO5087-stop-R	ggccgatacgggacccctctatgccggatccaccggcgcc
SCO5088-stop-F	gtggtcggcggtgctggacg
SCO5088-stop-R	cgtccagcaccgccgaccactacggcgccaggccgaggcc
SCO5089-stop-F	tagtgcgccggtgagacgga
SCO5089-stop-R	tccgtctcaccggcgcactacacgagggcgcggcgcagat
SCO5090-stop-F	taggccccggcgcggcggct
SCO5090-stop-R	agccgccgcgccggggcctaggtcctcagggtgtggacca
SCO5091-stop-F	tagggtgtctacgcctacga
SCO5091-stop-R	tcgtaggcgtagacaccctaggcgacctcacggacctcga
SCO5092-stop-F	gagtcccgcacggtttcacc
SCO5092-stop-R	ggtgaaaccgtgcgggactcacccgcggtcatgggcggtga
pUCK-Ori	agagcattacgctgacttga
pUCK-Kan	tcaagtcagcgtaatgctct

Table S4. Annotation of the *act* genes in *S. coelicolor*.

Gene	Gene Name	Annotation	Ref.
SCO5070	*act*VI-ORFB	hydroxylacyl-CoA dehydrogenase	2
SCO5071	*act*VI-ORFA	hydroxylacyl-CoA dehydrogenase	3
SCO5072	*act*VI-ORF1	hydroxylacyl-CoA dehydrogenase	4
SCO5073	*act*VI-ORF2	putative oxidoreductase	5
SCO5074	*act*VI-ORF3	putative dehydratase	2, 6
SCO5075	*act*VI-ORF4	putative oxidoreductase	2, 6
SCO5076	*act*VA-ORF1	integral membrane protein	7
SCO5077	*act*VA-ORF2	hypothetical protein	7
SCO5078	*act*VA-ORF3	hypothetical protein	8
SCO5079	*act*VA-ORF4	conserved hypothetical protein	8
SCO5080	*act*VA-ORF5	putative hydrolase	9
SCO5081	*act*VA-ORF6	hypothetical protein	9
SCO5082	*act*II-ORF1	putative transcriptional regulatory protein	10
SCO5083	*act*II-ORF2	putative actinorhodin transporter	10
SCO5084	*act*II-ORF3	putative membrane protein	10
SCO5085	*act*II-ORF4	actinorhodin cluster activator protein	11
SCO5086	*act*III	ketoacyl reductase	12
SCO5087	*act*I-ORF1	actinorhodin polyketide beta-ketoacyl synthase alpha subunit	13
SCO5088	*act*I-ORF2	actinorhodin polyketide beta-ketoacyl synthase beta subunit	13
SCO5089	*act*I-ORF3	actinorhodin polyketide synthase acyl carrier protein	14
SCO5090	*act*VII	actinorhodin polyketide synthase bifunctional cyclase/dehydratase	15
SCO5091	*act*IV	cyclase	15
SCO5092	*act*VB	flavin:NADH oxidoreductase	9, 16

Table S5. Mutations to permit *act* cluster assembly with BsaI and PaqCI.

Location	Type IIS	Mutation	Gene	Function
470	PaqCI	C → G	*act*VI-ORFB	hydroxylacyl-CoA dehydrogenase
1013	BsaI	G → C		
1290	BsaI	G → C		non-coding region
3013	BsaI	C → G	*act*VI-ORF1	hydroxylacyl-CoA dehydrogenase
3273	BsaI	A → C	*act*VI-ORF2	putative oxidoreductase
4066	BsaI	C → G		non-coding region
4419	BsaI	T → C	*act*VI-ORF3	putative dehydratase
6272	BsaI	C → G	*act*VA-ORF1	integral membrane protein
7368	BsaI	C → G	*act*VA-ORF2	hypothetical protein
7392	BsaI	C → G		
7849	BsaI	T → C	*act*VA-ORF3	hypothetical protein
10146	BsaI	T → C	*act*VA-ORF5	putative hydrolase
12304	BsaI	T → C	*act*II-ORF2	putative actinorhodin transporter
12828	PaqCI	T → G		
13525	BsaI	C → G		
14040	BsaI	T → C	*act*II-ORF3	putative actinorhodin transporter
14499	BsaI	T → C		
14907	BsaI	C → G		
16463	BsaI	C → G	*act*II-ORF4	actinorhodin cluster activator protein
16978	BsaI	A → G	*act*III	ketoacyl reductase
17155	BsaI	G → C		
18197	BsaI	C → G	*act*I-ORF1	actinorhodin polyketide beta-ketoacyl synthase alpha subunit
18566	BsaI	C → G		
18959	BsaI	C → G		
19522	BsaI	C → G	*act*I-ORF2	actinorhodin polyketide beta-ketoacyl synthase beta subunit (Chain Length Factor)
19564	BsaI	C → G	*act*I-ORF2	
20365	BsaI	C → G	*act*I-ORF3	actinorhodin polyketide synthase acyl carrier protein
22304	PaqCI	C → G	*act*IV	cyclase

Table S6. Sequence of synthetic promoter-RBS combination.

Promoter-RBS	Sequence (5' to 3')
SP2-RBS15	tgttcacattcgaaccgtctctgctttgacagagcccctaccagaattgtaaagtcgtggccatctaagtaaggagtgtccat
SP5-RBS15	tgttcacattcgaaccgtctctgctttgacatcttatgattctcgactgtaaagtcgtggccatctaagtaaggagtgtccat
SP10-RBS15	tgttcacattcgaaccgtctctgctttgacatgttcttacggtcacatgtaaagtcgtggccatctaagtaaggagtgtccat
SP15-RBS15	tgttcacattcgaaccgtctctgctttgacacctacgtgacacatcttgtaaagtcgtggccatctaagtaaggagtgtccat
SP20-RBS15	tgttcacattcgaaccgtctctgctttgacacatgacgctcacccgttgtaaagtcgtggccatctaagtaaggagtgtccat
SP26-RBS15	tgttcacattcgaaccgtctctgctttgacagctcactgggcatgggtgtaaagtcgtggccatctaagtaaggagtgtccat

Blue, synthetic promoter sequence; yellow, RBS sequence.

Table S7. Comparison of hierarchical GGA with other DNA assembly methods.

Feature	Hierarchical GGA	Gibson Assembly	RecET Recombineering	TAR cloning
**Core Mechanism**	Type IIS restriction enzymes + ligase in one-pot digestion–ligation cycles; predefined overhangs direct fragment order	Exonuclease, DNA polymerase, and ligase join DNA fragments with overlapping ends in a one-pot isothermal reaction	λ-phage derived recombinases (RecE/RecT) mediate homologous recombination between DNA with homology arms inside *E. coli*	In vivo homologous recombination in *Saccharomyces cerevisiae* assembles DNA fragments with long homology regions
**Homology Requirement**	Not needed; uses designed 4 bp overhangs	Requires 20–40 bp overlaps between adjacent fragments	Requires 80 bp homology arms for optimal efficiency	Requires ~40 bp to >1 kb homology arms
**Fragment Number**	10–20+ in a single reaction (hierarchical schemes allow unlimited scaling)	Up to 10 fragments; more fragments = lower efficiency	Typically one fragment from genomic DNA	Typically one fragment from genomic DNA
**Seamlessness**	Yes	Yes	Yes	Yes
**Refactoring Scalability**	Very high	Medium; overlaps must be redesigned each time	Low; case-specific	Low; case-specific
**In Vitro vs. In Vivo**	In vitro (enzyme mix)	In vitro (isothermal mix)	In vivo inside *E. coli*	In vivo inside *S. cerevisiae*
**Ease of Use**	Standardized	Straightforward for small to medium constructs	Requires engineering host strain with recombinase genes	Requires yeast expertise and handling large DNA
**Typical Applications**	Pathway assembly and large-scale refactoring	Cloning BGC, moderate-size pathway construction	Direct cloning of large BGCs	Direct cloning of large BGCs
**Limitations**	Reliant on removal of internal Type IIS sites; designing unique overhangs can be tricky	Efficiency decreases with fragment number and size; overlaps need PCR design	Requires specialized strains; limited throughput	Rearrangements in yeast, limited throughput
**BGC Modification**	Well-suited: allows modular swaps, promoter replacement, and multi-target mutations	Not feasible for multi-target/domain swaps	Usually single-locus modifications	Powerful for capturing intact BGCs, but needs to combine with CRISPR-Cas9 tools for precise edits
**Repetitive Sequences**	Well tolerant	Challenging	Challenging	Challenging

Reference

(1) Gomez-Escribano, J. P.; Bibb, M. J. Engineering *Streptomyces coelicolor* for heterologous expression of secondary metabolite gene clusters. *Microb. Biotechnol.* **2011**, *4* (2), 207-215.
(2) Hesketh, A.; Chater, K. F. Evidence from proteomics that some of the enzymes of actinorhodin biosynthesis have more than one form and may occupy distinctive cellular locations. *J. Ind. Microbiol. Biotechnol.* **2003**, *30* (8), 523-529.
(3) Zhu, X.; Wang, R.; Siitonen, V.; Vuksanovic, N.; Silvaggi, N. R.; Melançon III, C. E.; Metsä-Ketelä, M. ActVI-ORFA directs metabolic flux towards actinorhodin by preventing intermediate degradation. *PLoS One* **2024**, *19* (8), e0308684.
(4) Itoh, T.; Taguchi, T.; Kimberley, M. R.; Booker-Milburn, K. I.; Stephenson, G. R.; Ebizuka, Y.; Ichinose, K. Actinorhodin biosynthesis: structural requirements for post-PKS tailoring intermediates revealed by functional analysis of ActVI-ORF1 reductase. *Biochemistry* **2007**, *46* (27), 8181-8188.
(5) Ishikawa, K.; Hashimoto, M.; Komatsu, K.; Taguchi, T.; Okamoto, S.; Ichinose, K. Characterization of stereospecific enoyl reductase ActVI-ORF2 for pyran ring formation in the actinorhodin biosynthesis of *Streptomyces coelicolor* A3 (2). *Bioorganic Med. Chem. Lett.* **2022**, *66*, 128727.
(6) Hashimoto, M.; Ishikawa, K.; Fukushima, Y.; Shimazu, S.; Yabuzaki, M.; Kamezawa, Y.; Taguchi, T.; Ichinose, K. Characterization of ActVI-ORF3 and ActVI-ORF4 as Lactonizing and Delactonizing Enzymes in Relation to Metabolic Flux in Actinorhodin Biosynthesis. *ChemBioChem* **2025**, *26* (9), e202500049.
(7) Caballero, J. L.; Martinez, E.; Malpartida, F.; Hopwood, D. A. Organisation and functions of the *act*VA region of the actinorhodin biosynthetic gene cluster of *Streptomyces coelicolor*. *Mol. Genet. Genom.* **1991**, *230*, 401-412.
(8) Hashimoto, M.; Watari, S.; Taguchi, T.; Ishikawa, K.; Kumamoto, T.; Okamoto, S.; Ichinose, K. Actinorhodin Biosynthesis Terminates with an Unprecedented Biaryl Coupling Reaction. *Angew. Chem. Int. Ed.* **2023**, *62* (5). DOI: 10.1002/anie.202214400.
(9) Okamoto, S.; Taguchi, T.; Ochi, K.; Ichinose, K. Biosynthesis of actinorhodin and related antibiotics: discovery of alternative routes for quinone formation encoded in the *act* gene cluster. *Chem. Biol.* **2009**, *16* (2), 226-236.
(10) Tahlan, K.; Ahn, S. K.; Sing, A.; Bodnaruk, T. D.; Willems, A. R.; Davidson, A. R.; Nodwell, J. R. Initiation of actinorhodin export in *Streptomyces coelicolor*. *Mol. Microbiol.* **2007**, *63* (4), 951-961. DOI: 10.1111/j.1365-2958.2006.05559.x.
(11) Fujii, T.; Gramajo, H.; Takano, E.; Bibb, M. *redD* and *act*II-ORF4, pathway-specific regulatory genes for antibiotic production in *Streptomyces coelicolor* A3 (2), are transcribed in vitro by an RNA polymerase holoenzyme containing σ^{hrdD}. *J. Bacteriol.* **1996**, *178* (11), 3402-3405.
(12) Hadfield, A. T.; Limpkin, C.; Teartasin, W.; Simpson, T. J.; Crosby, J.; Crump, M. P. The crystal structure of the *act*III actinorhodin polyketide reductase: proposed mechanism for ACP and polyketide binding. *Structure* **2004**, *12* (10), 1865-1875.
(13) Kim, E.-S.; Hopwood, D. A.; Sherman, D. H. Analysis of type II polyketide β-ketoacyl synthase specificity in *Streptomyces coelicolor* A3 (2) by *trans* complementation of actinorhodin synthase mutants. *J. Bacteriol.* **1994**, *176* (6), 1801-1804.
(14) Revill, W. P.; Bibb, M. J.; Hopwood, D. A. Relationships between fatty acid and polyketide synthases from *Streptomyces coelicolor* A3 (2): characterization of the fatty acid synthase acyl carrier protein. *J. Bacteriol.* **1996**, *178* (19), 5660-5667.
(15) McDaniel, R.; Ebert-Khosla, S.; Hopwood, D. A.; Khosla, C. Engineered biosynthesis of novel polyketides: *act*VII and *act*IV genes encode aromatase and cyclase enzymes, respectively. *J. Am. Chem. Soc.* **1994**, *116* (24), 10855-10859.
(16) Filisetti, L.; Fontecave, M.; Nivière, V. Mechanism and substrate specificity of the flavin reductase ActVB from *Streptomyces coelicolor*. *J. Biol. Chem.* **2003**, *278* (1), 296-303.
